# International Society of Sports Nutrition position stand: sodium bicarbonate and exercise performance

**DOI:** 10.1186/s12970-021-00458-w

**Published:** 2021-09-09

**Authors:** Jozo Grgic, Zeljko Pedisic, Bryan Saunders, Guilherme G. Artioli, Brad J. Schoenfeld, Michael J. McKenna, David J. Bishop, Richard B. Kreider, Jeffrey R. Stout, Douglas S. Kalman, Shawn M. Arent, Trisha A. VanDusseldorp, Hector L. Lopez, Tim N. Ziegenfuss, Louise M. Burke, Jose Antonio, Bill I. Campbell

**Affiliations:** 1 Institute for Health and Sport, Victoria University, Melbourne, Australia; 2 Applied Physiology and Nutrition Research Group, School of Physical Education and Sport; Rheumatology Division; Faculdade de Medicina FMUSP, Universidade de Sao Paulo, Sao Paulo, SP, BR, University of São Paulo, Sao Paulo, Brazil; 3 Institute of Orthopaedics and Traumatology, Faculty of Medicine FMUSP, University of São Paulo, Sao Paulo, Brazil; 4 Centre for Bioscience, Manchester Metropolitan University, Manchester, M1 5GD UK; 5 Department of Health Sciences, Lehman College, Bronx, NY USA; 6 Exercise & Sport Nutrition Lab, Human Clinical Research Facility, Department of Health & Kinesiology, Texas A&M University, College Station, TX USA; 7 Physiology of Work and Exercise Response (POWER) Laboratory, Institute of Exercise Physiology and Rehabilitation Science, School of Kinesiology and Physical Therapy, University of Central Florida, Orlando, FL USA; 8 Nutrion Department, College of Osteopathic Medicine, Nova Southeastern University, Fort Lauderdale, FL 33314 USA; 9 Scientific Affairs. Nutrasource, Guelph, ON Canada; 10 Department of Exercise Science, Arnold School of Public Health, University of South Carolina, Columbia, SC USA; 11 Department of Exercise Science and Sport Management, Kennesaw State University, Kennesaw, GA USA; 12 The Center for Applied Health Sciences, Stow, OH USA; 13 Supplement Safety Solutions, Bedford, MA 01730 USA; 14 Exercise and Nutrition Research Program, Mary MacKillop Institute for Health Research, Australian Catholic University, Melbourne, Australia; 15 Exercise and Sport Science, Nova Southeastern University, Davie, FL 33314 USA; 16 Performance & Physique Enhancement Laboratory, University of South Florida, Tampa, FL 33612 USA

## Abstract

Based on a comprehensive review and critical analysis of the literature regarding the effects of sodium bicarbonate supplementation on exercise performance, conducted by experts in the field and selected members of the International Society of Sports Nutrition (ISSN), the following conclusions represent the official Position of the Society:

1. Supplementation with sodium bicarbonate (doses from 0.2 to 0.5 g/kg) improves performance in muscular endurance activities, various combat sports, including boxing, judo, karate, taekwondo, and wrestling, and in high-intensity cycling, running, swimming, and rowing. The ergogenic effects of sodium bicarbonate are mostly established for exercise tasks of high-intensity that last between 30 s and 12 min.
2. Sodium bicarbonate improves performance in single- and multiple-bout exercise.
3. Sodium bicarbonate improves exercise performance in both men and women.
4. For single-dose supplementation protocols, 0.2 g/kg of sodium bicarbonate seems to be the minimum dose required to experience improvements in exercise performance. The optimal dose of sodium bicarbonate dose for ergogenic effects seems to be 0.3 g/kg. Higher doses (e.g., 0.4 or 0.5 g/kg) may not be required in single-dose supplementation protocols, because they do not provide additional benefits (compared with 0.3 g/kg) and are associated with a higher incidence and severity of adverse side-effects.
5. For single-dose supplementation protocols, the recommended timing of sodium bicarbonate ingestion is between 60 and 180 min before exercise or competition.
6. Multiple-day protocols of sodium bicarbonate supplementation can be effective in improving exercise performance. The duration of these protocols is generally between 3 and 7 days before the exercise test, and a total sodium bicarbonate dose of 0.4 or 0.5 g/kg per day produces ergogenic effects. The total daily dose is commonly divided into smaller doses, ingested at multiple points throughout the day (e.g., 0.1 to 0.2 g/kg of sodium bicarbonate consumed at breakfast, lunch, and dinner). The benefit of multiple-day protocols is that they could help reduce the risk of sodium bicarbonate-induced side-effects on the day of competition.
7. Long-term use of sodium bicarbonate (e.g., before every exercise training session) may enhance training adaptations, such as increased time to fatigue and power output.
8. The most common side-effects of sodium bicarbonate supplementation are bloating, nausea, vomiting, and abdominal pain. The incidence and severity of side-effects vary between and within individuals, but it is generally low. Nonetheless, these side-effects following sodium bicarbonate supplementation may negatively impact exercise performance. Ingesting sodium bicarbonate (i) in smaller doses (e.g., 0.2 g/kg or 0.3 g/kg), (ii) around 180 min before exercise or adjusting the timing according to individual responses to side-effects, (iii) alongside a high-carbohydrate meal, and (iv) in enteric-coated capsules are possible strategies to minimize the likelihood and severity of these side-effects.
9. Combining sodium bicarbonate with creatine or beta-alanine may produce additive effects on exercise performance. It is unclear whether combining sodium bicarbonate with caffeine or nitrates produces additive benefits.
10. Sodium bicarbonate improves exercise performance primarily due to a range of its physiological effects. Still, a portion of the ergogenic effect of sodium bicarbonate seems to be placebo-driven.

## Introduction

Sodium bicarbonate is used as an ergogenic aid and as an ingredient in prescription and over-the-counter medications [1]. Many studies have explored the effects of sodium bicarbonate on performance in various modes of exercise, including combat sport tasks, resistance exercise, and single and repeated high-intensity cycling, running, swimming, and rowing (Table 1) [2–125]. The effects of different sodium bicarbonate ingestion protocols to maximize the ergogenic effects while minimizing the incidence and severity of side-effects have also been examined [38, 46, 47, 55, 75, 77, 99, 121]. Studies have also investigated the interaction of sodium bicarbonate with other ergogenic aids, such as beta-alanine, caffeine, and creatine (Table 2) [126, 127, 129–147]. The purpose of this position stand was to: (1) critically evaluate and summarize the scientific literature on the ergogenic effects of sodium bicarbonate; (2) provide recommendations for the use of sodium bicarbonate as an ergogenic aid; and (3) suggest key topics for future research on sodium bicarbonate supplementation.

**Table 1:** Summary of studies exploring the effects of sodium bicarbonate on exercise performance

Study	Participants	Exercise performance test	Sodium bicarbonate dose	Timing of ingestion	Main findings
Afman et al. 2014 [2]	7 well-trained male basketball players	Basketball layups and 15-m sprints in 4 quarters	0.4 g/kg	90 to 20 min before exercise	Made layups: ↔ Sprint time: ↓ in the 4^th^ quarter
Ansdell et al. 2020 [3]	10 male basketball players	Isometric knee extension after each quarter of a basketball game, 15-m sprint, and basketball layups	0.4 g/kg	90 to 60 min before exercise	Made layups: ↔ Sprint time: ↔ MVC: sodium bicarbonate ingestion attenuated the decline in strength
Artioli et al. 2007 [4]	23 judo competitors	3 × SJFT (5-min rest); 4 × modified upper body Wingate test (3-min rest)	0.3 g/kg	120 min before exercise	Number of throws: ↑ in SJFT bout 2 and 3 Mean power: ↑ in Wingate bout 3 and 4 Peak power: ↑ in Wingate bout 4
Aschenbach et al. 2000 [5]	8 college wrestlers	8 × 15-s arm ergometer (20-s rest)	0.3 g/kg	90 to 60 min before exercise	Peak power: ↔ Total work: ↔
Bird et al. 1995 [6]	12 male distance runners	1500-m run	0.3 g/kg	120 to 60 min before exercise	Running time: ↓
Bishop et al. 2004 [7]	10 female team-sport athletes	5 × 6-s cycle sprints (30-s rest)	0.3 g/kg	90 min before exercise	Total work: ↑ Peak power: ↑ in sprints 3, 4, and 5
Bishop et al. 2005 [8]	7 female team-sport athletes	2 × 36-min “halves” of 18 × 4-s cycle sprints (2-min rest)	0.4 g/kg	90 to 20 min before exercise	Work: ↑ in 7 of 18 second-half sprints Peak power: ↑ in 8 of 18 second-half sprints
Boegman et al. 2020 [9]	23 male rowers	2000-m rowing	0.3 g/kg	60 min before exercise, or according to individualized ingestion timing	Rowing time: ↓ with the individualized protocol
Bouissou et al. 1988 [10]	6 male varsity track athletes	Cycling to exhaustion at 125% of peak aerobic work	0.3 g/kg	120 min before exercise	Time to fatigue: ↑
Brien et al. 1989 [11]	6 well-trained oarsmen	4-min submaximal rowing and 2-min maximum rowing	0.3 g/kg	180 to 60 min before exercise	Work: ↔ Power: ↔
Brisola et al. 2015 [12]	15 moderately active males	2 × supramaximal running at 110% of VO_2max_	0.3 g/kg	90 min before exercise	Time to fatigue: ↔
Cameron et al. 2010 [13]	25 male rugby players	Rugby-specific repeated-sprint test	0.3 g/kg	90 min before exercise	Mean sprint time: ↔ Fastest 40-m sprint time: ↔ Mean change in repeated-sprint ability: ↔
Campos et al. 2012 [14]	3 female and 7 male swimmers	6 × 100-m swimming (6-min of rest)	0.3 g/kg	60 min before exercise	Stroke length: ↔ Stroke rate: ↔ Stroke index: ↔ Swimming time: ↔
Carr et al. 2012 [15]	3 female and 4 male well-trained rowers	2000-m rowing	Acute protocol: 0.3 g/kg Multiple-day protocol: 0.5 g/kg	Acute protocol: 120 min before exercise Multiple-day protocol: for 3 days before exercise	Mean power: ↔
Carr et al. 2013 [16]	12 resistance-trained men	4 sets of squat and leg press, and 5 sets of knee extensions using 10-12RM or 50% of 1RM (60-s to 90-s rest)	0.3 g/kg	80 to 50 min before exercise	Number of repetitions: ↑
Cholewa et al. 2015 [17]	7 division III collegiate male soccer players	Yo-Yo intermittent recovery test level 2	0.3 g/kg	60 min before exercise	Distance covered: ↔ Time to fatigue: ↔
Coombes et al. 1993 [18]	9 male physical education students	Isokinetic knee extension and flexion	0.3 g/kg	90 min before exercise	Peak torque: ↑ Total work: ↑
Costill et al. 1984 [19]	1 female and 10 males	4 × 1-min cycling bout (1-min rest), 5^th^ sprint was to exhaustion	0.2 g/kg	60 min before exercise	Time to fatigue: ↑
Dalle et al. 2019 [20]	12 physically active males	4 × 2-min cycling (each bout was separated by a 3-h rest interval)	0.4 g/kg	Over a 9 h period before exercise	Mean power: ↑ in bout 3
Dalle et al. 2021 [21]	11 male cyclists	90-s sprint at the end of 3-h simulated cycling race	0.3 g/kg	From 120 min before exercise until the end of the 3 h simulated cycling race	Mean power: ↑
Deb et al. 2017 [22]	11 male trained cyclists	3-min all-out cycling performed in normoxia and hypoxia	0.3 g/kg	According to individualized ingestion timing	Curvature constant: ↑ in normoxia and hypoxia Critical power: ↔ Peak power: ↔ Total work: ↑ in normoxia and hypoxia
Deb et al. 2018 [23]	11 recreationally active males	Repeated 60-s cycling to exhaustion (30-s rest)	0.3 g/kg	According to individualized ingestion timing	Exercise tolerance: ↑ Work done: ↑
Delextrat et al. 2018 [24]	15 female basketball players	Basketball Exercise Simulated Test	0.3 g/kg	For 4 days before exercise	Mean sprint time: ↓ Mean circuit time: ↓ Mean jump height: ↑ Ideal sprint time: ↓ Total sprint time: ↓ Sprint performance decrement: ↓ Jump height decrement: ↔
Dixon et al. 2017 [25]	8 recreationally active males	Yo-Yo intermittent recovery test 1	0.3 g/kg	60 min before exercise	Time to fatigue: ↑
Douroudos et al. 2006 [26]	24 men (8 in each group)	Wingate test	0.3 or 0.5 g/kg	For 5 days before exercise	Mean power: ↑ only with 0.5 g/kg
Driller et al. 2012 [27]	8 male cyclists	4-min cycling	Acute protocol: 0.3 g/kg Multiple-day protocol: 0.4 g/kg	Acute protocol: 90 to 30 min before exercise Multiple-day protocol: for 3 days before exercise	Absolute mean power: ↑ with both protocols Relative mean power: ↑ only with the acute protocol
Driller et al. 2012 [28]	8 well-trained male cyclists	2-min cycling	0.3 g/kg	120 to 60 min before exercise	Mean power: ↑ Peak power: ↔
Driller et al. 2013 [29]	12 Australian representative rowers	2000-m rowing	0.3 g/kg	90 min before every exercise session (4 weeks, 2 days per week)	Rowing time: ↔ Mean power: ↔ Peak power: ↔ Power at 4 mmol/L of lactate: ↔
Duncan et al. 2014 [30]	8 resistance-trained men	3 sets of squat and bench press at 80% of 1RM (3-min rest)	0.3 g/kg	60 min before exercise	Number of repetitions in the squat: ↑ Number of repetitions in the bench press: ↔
Durkalec-Michalski et al. 2018 [31]	9 female and 12 male CrossFit-trained participants	3 × Fight Gone Bad CrossFit round and incremental cycling test	0.037.5 to 0.150 g/kg	For 10 days before exercise	Number of repetitions: ↑ Time to exhaustion: ↔ Maximum workload: ↔ Workload at ventilatory threshold: ↑ Time to ventilatory threshold: ↑
Durkalec-Michalski et al. 2018 [32]	18 female and 31 male wrestlers	2 × Wingate test with a dummy throw test during the rest interval	0.025 to 0.150 g/kg	For 10 days before exercise	Peak power: ↔ Mean power: ↔ Minimum power: ↔ Time-to-peak power: ↓ Number of throws: ↔
Durkalec-Michalski et al. 2020 [33]	24 male field hockey players	2 × Wingate test with a discipline-specific field performance test during the rest interval	Acute protocol: 0.2 g/kg Multiple-day protocol: 0.05 to 0.2 g/kg	Acute protocol: 120 min before exercise Multiple-day protocol: for 8 days before exercise	Work: ↑ in Wingate bout 1 only with multiple-day protocol Mean power: ↑ in Wingate bout 1 only with multiple-day protocol Peak power: ↑ in Wingate bout 1 only with multiple-day protocol Time-to-peak power: ↔ Power carry threshold at 97% of peak power: ↑ in Wingate bout 1 only with multiple-day protocol Average power at power carry threshold: ↑ in Wingate bout 1 only with multiple-day protocol Time needed to complete the discipline-specific performance test: ↓ with both protocols
Durkalec-Michalski et al. 2020 [34]	18 female and 33 male wrestlers	2 × Wingate test with a dummy throw test during the rest interval	0.025 to 0.150 mg/kg	For 10 days before exercise	Peak power: ↔ Mean power: ↔ Power drop: ↔ Power in the middle section of the Wingate test: ↑ Number of throws: ↑ only in males
Edge et al. 2006 [35]	16 recreationally active women	Constant-load exercise test	0.4 g/kg	90 to 30 min before every exercise session (8 weeks, 3 days per week)	Time to fatigue: significantly greater improvements in the group supplementing with sodium bicarbonate
Egger et al. 2014 [36]	21 well-trained cyclists	Stepwise incremental exercise tests and 30-min at 95% of the individual anaerobic threshold followed by 110% at individual anaerobic threshold until exhaustion	0.3 g/kg	60 min before exercise	Maximal workload: ↔ Workload at 110% of individual anaerobic threshold: ↔ Time to fatigue: ↑
Farney et al. 2020 [37]	11 recreationally trained men	Isometric mid-thigh pull after an exercise session consisting of barbell thrusters, squat jumps, lunge jumps, and forward jumps	0.3 g/kg	60 min before exercise	Peak force: ↔ Rate of force development: ↔
Ferreira et al. 2019 [38]	21 male cyclists	Cycling to exhaustion	0.1 or 0.3 g/kg	30 min before exercise	Time to fatigue: ↑ only with 0.3 g/kg
Freis et al. 2017 [39]	18 trained runners	Graded exercise test and 30-min at 95% of the individual anaerobic threshold followed by 110% at individual anaerobic threshold until exhaustion	0.3 g/kg	90 to 30 min before exercise	Time to fatigue: ↔ Maximum running speed: ↑
Froio De Araujo Dias et al. 2015 [40]	15 physically active males	Cycling capacity test at 110% of maximum power output	0.3 g/kg	90 min before exercise (same protocol was repeated 4 times)	Total work: ↑ only in 1 out of the 4 testing sessions
Gaitanos et al. 1991 [41]	7 males	10 × 6-s all-out running sprints (30-s rest)	0.3 g/kg	150 min before exercise	Mean power: ↔ Peak power: ↔ Mean running speed: ↔ Peak running speed: ↔
Gao et al. 1988 [42]	10 well-trained male swimmers	5 × 91.4 m swimming (2-min rest)	0.29 g/kg	60 min before exercise	Swimming velocity: ↑ in swimming bouts 4 and 5
George et al. 1988 [43]	7 males involved in competitive sports	Continuous running incremental test	0.2 g/kg	150 to 90 min before exercise	Time to fatigue: ↑
Goldfinch et al. 1988 [44]	6 trained male athletes	400-m running	0.4 g/kg	60 min before exercise	Running time: ↓
Gough et al. 2017 [45]	9 active males	2 × cycling to exhaustion at peak mean minute power (90-min rest)	0.3 g/kg	30 min after the first cycling test	Time to fatigue: ↑
Gough et al. 2017 [46]	11 trained male cyclists	4-km cycling time trial	0.2 or 0.3 g/kg	According to individualized ingestion timing (both protocols were repeated twice)	Cycling time: ↓ with 0.2 or 0.3 g/kg
Gough et al. 2018 [47]	10 male trained cyclists	2 × 4-km cycling time trial (40-min rest)	0.2 or 0.3 g/kg	According to individualized ingestion timing	Cycling time: ↓ in bout 1 and 2 only with 0.3 g/kg
Gough et al. 2018 [48]	11 trained male cyclists	4-km cycling time trial	0.2 or 0.3 g/kg	According to individualized ingestion timing	Cycling time: ↓ with 0.2 or 0.3 g/kg
Gough et al. 2019 [49]	7 elite male professional boxers	Boxing specific high-intensity interval running protocol, followed by 2 × high-intensity running to volitional exhaustion (75-min rest) and boxing specific punch combination protocol	0.3 g/kg	65 min after the first high-intensity run to exhaustion	Time to fatigue: ↑
Gough et al. 2019 [50]	1 female and 13 male club level cyclists	4-km cycling time trial in hypoxia	0.2 or 0.3 g/kg	According to individualized ingestion timing	Cycling time: ↓ with 0.2 or 0.3 g/kg
Gurton et al. 2020 [51]	12 recreationally active males	3 × 60-s cycling at 90%, 95%, and 100% of maximal aerobic power (90-s rest), followed by cycling to exhaustion at 105% of maximal aerobic power	0.2 or 0.3 g/kg	60 min before exercise	Cycling time: ↓ with 0.2 or 0.3 g/kg Mean power: ↑ with 0.2 or 0.3 g/kg Mean speed: ↔
Higgins et al. 2013 [52]	10 non-cycling trained males	Cycling to exhaustion at 100%, 110% and 120% peak mean minute power	0.3 g/kg	60 min before exercise	Time to fatigue at 100%: ↑ Time to fatigue at 110%: ↔ Time to fatigue at 120%: ↔
Hilton et al. 2020 [53]	11 trained male cyclists	4-km cycling time trial	0.3 g/kg	According to individualized ingestion timing (enteric-coated or gelatin capsules)	Cycling time: ↓ with enteric-coated and gelatin capsules Mean power: ↑ only with gelatin capsules
Hobson et al. 2014 [54]	20 male rowers	2000-m rowing	0.3 g/kg	240 to 120 min before exercise	Rowing time: ↓
Horswill et al. 1988 [55]	9 endurance-trained cyclists	2-min cycling sprints	0.1, 0.15, or 0.2 g/kg	60 min before exercise	Total work: ↔
Inbar et al. 1983 [56]	13 male physical education students	Wingate test	10 g	180 min before exercise	Mean power: ↑ Peak power: ↔
Iwaoka et al. 1989 [57]	6 male physical education students	Cycling to exhaustion at 95% VO_2max_	0.2 g/kg	120 min before exercise	Time to fatigue: ↑
Jones et al. 1977 [58]	5 males	Cycling at 95% of peak power output	0.3 g/kg	180 min before exercise	Time to fatigue: ↑
Joyce et al. 2012 [59]	8 highly trained male swimmers	200-m swimming (repeated on consecutive days)	Acute protocol: 0.3 g/kg Multiple-day protocol: 0.3 g/kg	Acute protocol: 120 to 90 min before exercise Multiple-day protocol: for 3 days before exercise	Swimming time: ↔
Katz et al. 1984 [60]	8 males	Cycling to exhaustion at 125% of VO_2max_	0.2 g/kg	60 min before exercise	Time to fatigue: ↔
Kowalchuk et al. 1984 [61]	6 males	Continuous incremental cycling test	0.3 g/kg	180 min before exercise	Time to fatigue: ↔ Peak power: ↔
Kozak-Collins et al. 1994 [62]	7 female cyclists	Repeated cycling sprints at 95% of VO_2max_ (1-min rest)	0.3 g/kg	120 to 105 min before exercise	Number of sprints: ↔
Krustrup et al. 2015 [63]	13 trained males	Yo-Yo intermittent recovery test level 2	0.4 g/kg	90 to 50 min before exercise	Distance covered: ↑
Kumstát et al. 2018 [64]	6 nationally ranked male swimmers	400-m swimming	0.3 g/kg	60 min before exercise	Swimming time: ↔
Lavender et al. 1989 [65]	8 females and 15 males	10 × 10-s cycle sprints (50-s rest)	0.3 g/kg	120 to 60 min before exercise	Mean power: ↑ in 8 out of 10 sprints Peak power: ↑ in 2 out of 10 sprints
Limmer et al. 2020 [66]	2 female and 8 male recreationally active participants	Portable tethered sprint running	0.3 g/kg	For 7 days before exercise	Peak force: ↔ Mean force: ↔
Lindh et al. 2008 [67]	9 male elite-standard swimmers	200-m swimming	0.3 g/kg	90 to 60 min before exercise	Swimming time: ↓
Lopes-Silva et al. 2018 [68]	9 male taekwondo black belt athletes	3 × 2-min taekwondo combat (1-min rest)	0.3 g/kg	90 min before exercise	Total attack time: ↑ Attack time: ↔ Stepping time: ↔ Pause time: ↔ Stepping time sum: ↔ Pause time sum: ↔ Attack number: ↔ Stepping number: ↔ Pause number: ↔
Marriot et al. 2015 [69]	12 male team-sport athletes	Arm-cranking exercise followed by Yo-Yo intermittent recovery test level 2	0.3 g/kg	90 to 60 min before exercise	Distance covered: ↑
Marx et al. 2002 [70]	10 males	90-s maximal cycle test	0.3 g/kg	65 to 20 min before exercise	Mean power: ↔ Peak power: ↔
Materko et al. 2008 [71]	11 resistance-trained men	Bench press and pull press 10RM	0.3 g/kg	120 min before exercise	Weight lifted: ↔
Matsuura et al. 2007 [72]	8 male undergraduate students	10 × 10-s cycle sprints (30-s or 360-s rest)	0.3 g/kg	180 to 120 min before exercise	Mean power: ↔ Peak power: ↔
Maughan et al. 1986 [73]	10 males	Isometric knee extension	0.3 g/kg	180 min before exercise	MVC: ↔ Time to maintain 20% of MVC: ↑ Time to maintain 50% of MVC: ↔ Time to maintain 80% of MVC: ↔
McCartney et al. 1983 [74]	6 males	Constant-velocity cycling for 30 s	0.3 g/kg	180 min before exercise	Mean power: ↔ Peak power: ↔ Total work: ↔ Rate of mean power decline: ↔
McKenzie et al. 1986 [75]	6 males athletes	5 × 1-min cycling (1-min rest), 6^th^ sprint was to exhaustion	0.15 or 0.3 g/kg	60 min before exercise	Time to fatigue: ↑ with 0.15 or 0.3 g/kg
McNaughton et al. 1991 [76]	8 trained male cyclists	60-s cycling	0.4 g/kg	60 min before exercise	Total work: ↑ Peak power: ↑
McNaughton 1992 [77]	9 males	60-s cycling	0.1, 0.2, 0.3, 0.4, or 0.5 g/kg	60 min before exercise	Total work: ↑ with doses from 0.2 to 0.5 g/kg Peak power: ↑ with doses from 0.3 to 0.5 g/kg
McNaughton 1992 [78]	32 males (8 in each group)	10-s, 30-s, 120-s, or 240-s cycling	0.3 g/kg	90 min before exercise	Total work 10-s cycling: ↔ Total work 30-s cycling: ↔ Total work 120-s cycling: ↑ Total work 240-s cycling: ↑ Peak power 10-s cycling: ↔ Peak power 30-s cycling: ↔ Peak power 120-s cycling: ↑ Peak power 240-s cycling: ↑
McNaughton et al. 1997 [79]	10 moderately trained females	60-s cycling	0.3 g/kg	90 min before exercise	Total work: ↑ Peak power: ↑
McNaughton et al. 1999 [80]	10 well-trained men	1-h cycling	0.3 g/kg	90 min before exercise	Mean power: ↑ Peak power: ↔ Total work: ↑
McNaughton et al. 1999 [81]	7 participants	60-s cycling	0.5 g/kg	For 5 days before exercise	Peak power: ↑ Total work: ↑
McNaughton et al. 2000 [82]	2 females and 6 males	3 × Wingate test (30-s rest)	0.5 g/kg	For 5 days before exercise	Peak power: ↑ in Wingate bout 1 and 2 Total work: ↑ in Wingate bout 1 and 2
McNaughton et al. 2001 [83]	8 males	90-s cycling on three consecutive days	Acute protocol: 0.5 g/kg Multiple-day protocol: 0.5 g/kg	Acute protocol: 90 min before exercise Multiple-day protocol: for 5 days before exercise	Total work day 1: ↑ with both protocols Total work day 2: ↑ only with multiple-day protocol Total work day 3: ↑ only with multiple-day protocol
Miller et al. 2016 [84]	11 male active team and individual sport athletes	10 × 6-s sprints (60-s rest)	0.3 g/kg	According to individualized ingestion timing	Total work: ↑ Peak power: ↔
Mündel 2018 [85]	10 male team sport athletes	2 × Wingate test (5-min rest)	0.5 g/kg	Ingested at 4 h intervals starting at 9 h before exercise	Total work: ↑ in Wingate bout 2 Peak power: ↔
Mueller et al. 2013 [86]	8 trained male cyclists and triathletes	Cycling time to exhaustion at critical power	0.3 g/kg	90 min before exercise (same protocol was repeated 5 times)	Time to fatigue: ↑ in all 5 testing sessions
Northgraves et al. 2014 [87]	7 recreationally active men	40-km cycling time trial	0.3 g/kg	60 min before exercise	Cycling time: ↔
Oliveira et al. 2017 [88]	18 male rugby, judo, and jiu-jitsu athletes	4 × modified upper body Wingate test (3-min rest)	0.5 g/kg	For 5 days before exercise	Total work (all bout): ↑ Total work Wingate bout 1: ↔ Total work Wingate bout 2: ↔ Total work Wingate bout 3: ↑ Total work Wingate bout 4: ↑
Parry-Billings et al. 1986 [89]	6 males	3 × Wingate test (6-min rest)	0.3 g/kg	150 min before exercise	Mean power: ↔ Peak power: ↔
Peart et al. 2011 [90]	7 physically active males	4-min cycling	0.3 g/kg	90 min before exercise	Total work: ↔ Mean power: ↔ Peak power: ↔
Peinado et al. 2018 [91]	12 elite male BMX cyclists	3 × BMX Olympic track races (15-min rest)	0.3 g/kg	90 min before exercise	Event time: ↔ Time to peak velocity: ↔ Peak velocity: ↔
Pierce et al. 1992 [92]	7 male varsity swimmers	91.4-m and 182.8-m swimming	0.2 g/kg	60 min before exercise	Swimming time: ↔
Potteiger et al. 1996 [93]	7 male runners	30-min treadmill running at the lactate threshold followed by running to exhaustion at 110% of lactate threshold	0.3 g/kg	120 min before exercise	Running time: ↔
Price et al. 2003 [94]	8 aerobically trained men	10 × 14-s cycle sprints (166-s rest)	0.3 g/kg	60 min before exercise	Peak power: sodium bicarbonate ingestion attenuated the decline in peak power
Price et al. 2010 [95]	8 aerobically trained men	20 × 24-s sprints (36 to 60-s rest) followed by running to exhaustion at velocity during 120% of VO_2max_	0.3 g/kg	60 min before exercise	Running time: ↔ Running distance: ↔
Raymer et al. 2004 [96]	6 males	Wrist flexion to fatigue	0.3 g/kg	90 min before exercise	Time to fatigue: ↑
Robertson et al. 1987 [97]	10 male university students	Ergometer tasks for upper-body, lower-body, or combined upper and lower-body	0.3 g/kg	120 to 105 min before exercise	Total work: ↑ in all exercise tests
Saunders et al. 2014 [98]	21 recreationally active males	Cycling capacity test at 110% of maximum power output	0.3 g/kg	240 to 120 min before exercise	Total work: ↑ only when considering data among the participants that did not experience side-effects
Schauf et al. 1996 [99]	12 moderately trained females	Repeated 1-min cycle at 110% VO_2peak_ to exhaustion (9-min rest)	0.1 g/kg or 0.2 g/kg	60 min before exercise	Time to fatigue: ↑ only with 0.2 g/kg
Siegler et al. 2010 [100]	10 amateur boxers	4 × 3-min boxing rounds (1-min rest)	0.3 g/kg	90 min before exercise	Punch efficacy: ↑
Siegler et al. 2010 [101]	9 active males	3 × 30-s runs (3-min rest)	0.3 g/kg	75 to 60 min before exercise	Average running speed: ↑ in bout 3 Peak running speed: ↔
Siegler et al. 2010 [102]	8 female and 6 male swimmers	8 × 25-m swimming (5-s rest)	0.3 g/kg	150 min before exercise	Swimming time: ↓
Siegler et al. 2012 [103]	8 recreationally active males	10 × 10-s sprints (50-s rest)	0.3 g/kg	60, 120, or 180 min before exercise	Average speed: ↔ Average power: ↔ Total distance: ↔
Siegler et al. 2013 [104]	2 female and 8 male cyclists	30-s sprints at 120% peak power output until exhaustion (30-s rest) and isometric knee extension	0.3 g/kg	90 to 30 min before exercise	Time to fatigue: ↔ MVC: ↔ Rate of force development: ↑
Siegler et al. 2015 [105]	8 recreationally active males	Submaximal calf contractions at 55 % of MVC, and MVC during the rest interval	0.3 g/kg	90 to 30 min before exercise	Time to fatigue: ↔ MVC: ↔
Siegler et al. 2015 [106]	11 recreationally active men	Knee extension with and without cuffs	0.3 g/kg	90 to 30 min before exercise	MVC: ↔ Rate of torque development: ↑
Siegler et al. 2016 [107]	10 resistance-trained men	Plantar flexion and elbow extension	0.3 g/kg	90 to 30 min before exercise	MVC: ↔ Rate of torque development: ↔
Siegler et al. 2018 [108]	6 male and 2 female resistance-trained participants	Knee extension	0.3 g/kg	90 to 30 min before exercise (acute protocol) and before every exercise session (10 weeks, 2 days per week)	Number of repetitions: ↔ MVC: ↔ 1RM: ↔ Rate of torque production: ↑
Sostaric et al. 2006 [109]	4 untrained females and 5 untrained males	Finger flexion	0.3 g/kg	180 to 120 min before exercise	Time to fatigue: ↑ Mean force: ↔ Mean power: ↔
Stephens et al. 2002 [110]	7 endurance-trained men	30-min of cycling exercise followed by ~30-min of cycling at ~80% of VO_2peak_	0.3 g/kg	120 to 90 min before exercise	Cycling time: ↔
Sutton et al. 1981 [111]	5 males	Cycling at 95% of peak power output	0.3 g/kg	180 min before exercise	Time to fatigue: ↑
Tan et al. 2010 [112]	12 elite players from the Australian National Women’s Water Polo Squad	56 × 10-m maximal-sprint swimming (17-s to 5-min rest)	0.3 g/kg	90 min before exercise	Mean sprint time: ↔
Tanaka et al. 2018 [113]	6 male students	Yo-Yo intermittent recovery test level 2	0.2 g/kg	60 to 20 min before exercise	Distance covered: ↔
Van Montfoort et al. 2004 [114]	15 competitive male endurance runners	Running to exhaustion	0.3 g/kg	180 to 90 min before exercise	Time to fatigue: ↑
Vanhatalo et al. 2010 [115]	8 habitually active males	3-min all-out cycling	0.3 g/kg	60 min before exercise	Total work: ↔ Work done above critical power: ↔
Verbitsky et al. 1997 [116]	6 males	Cycling at 117% of VO_2max_ followed by functional electrical stimulation	0.4 g/kg	60 min before exercise	Normalized torque: ↑
Wang et al. 2019 [117]	20 college-age males	Wingate test	0.2 g/kg	90 to 60 min before every exercise session (6 weeks, 3 days per week)	Mean power: ↔ Peak power: significantly greater improvements in the group supplementing with sodium bicarbonate
Webster et al. 1993 [118]	6 resistance-trained men	5 sets of leg press at 70% of 1RM (90-s rest), 5^th^ set was to failure	0.3 g/kg	105 min before exercise	Number of repetitions: ↔
Wilkes et al. 1983 [119]	6 male varsity track athletes	800-m running	0.3 g/kg	120 min before exercise	Running time: ↓
Wu et al. 2010 [120]	9 male college tennis players	2 × Loughborough Tennis Skill Tests and stimulated tennis match	0.3 g/kg	70 min before the first Loughborough Tennis Skill Tests and 0.1 g/kg ingested immediately before the second Loughborough Tennis Skill Tests	Service consistency: ↑ Ground stroke forehand: ↑ Accuracy of service: ↔ Ground stroke total: ↔ Ground stroke backhand: ↔
Yong et al. 2018 [121]	8 male swimmers	200-m swimming	0.2 or 0.3 g/kg	90 min before exercise	Swimming time: ↓ with 0.2 or 0.3 g/kg
Zabala et al. 2008 [122]	9 elite male BMX riders	3 × Wingate test (30-min rest)	0.3 g/kg	90 min before exercise	Peak power: ↔ Time to peak power: ↔ Mean power: ↔
Zabala et al. 2011 [123]	10 elite male BMX riders	3 × Wingate test (15-min rest)	0.3 g/kg	90 min before exercise	Peak power: ↔ Time to peak power: ↔ Mean power: ↔
Zajac et al. 2009 [124]	8 youth male swimmers	4 × 50-m swimming (1-min rest)	0.3 g/kg	90 min before exercise	Swimming speed: ↑ in bout 1 Swimming time: ↓
Zinner et al. 2011 [125]	11 aerobically well-trained men	4 × Wingate test (5-min rest)	0.3 g/kg	90 min before exercise	Mean power: ↑ in Wingate bouts 3 and 4 Peak power: ↔

BMX: bicycle motocross; MVC: maximal voluntary contraction; RM: repetition maximum; SJFT: Special Judo Fitness Test; VO_2max_: maximal oxygen uptake; VO_2peak_: peak oxygen uptake; ↑: significant increase; ↓: significant decrease; ↔: no significant difference

**Table 2:** Summary of studies exploring the effects of sodium bicarbonate in combination with beta-alanine, caffeine, creatine, and nitrates on exercise performance

Study	Participants	Exercise performance test	Sodium bicarbonate supplementation protocol	Additional supplement	Main findings
Barber et al. 2013 [126]	13 aerobically-trained males	10 × 10-s cycle sprints (60-s rest)	0.5 g/kg ingested for 2 days before exercise	Creatine: 20 g ingested for 2 days before exercise	Relative peak power: ↑ following isolated creatine ingestion and combined sodium bicarbonate and creatine ingestion Absolute peak power: ↑ following combined sodium bicarbonate and creatine ingestion Relative mean power: ↑ following combined sodium bicarbonate and creatine ingestion Absolute mean power: ↔
Bellinger et al. 2012 [127]	14 highly trained male cyclists	4-min cycling	0.3 g/kg ingested from 150 to 90 min before exercise	Beta-alanine: 6.5 g/kg ingested for 28 days before exercise	Mean power: ↑ following isolated sodium bicarbonate ingestion and combined sodium bicarbonate and beta-alanine ingestion Total work: ↑ following isolated sodium bicarbonate ingestion and combined sodium bicarbonate and beta-alanine ingestion
Callahan et al. 2017 [128]	8 male cyclists	4 × 4-km cycling time trial	0.3 g/kg ingested from 150 to 75 min before exercise	Beetroot crystals: 15 g/day for 3 days and 15 g 60 min before exercise	Mean power: ↔ Cycling time: ↔
Carr et al. 2011 [129]	2 female and 6 male well-trained rowers	2000-m rowing	0.3 g/kg ingested 90 min before exercise	Caffeine: 6 mg/kg ingested 30 min before exercise	Mean power: ↑ only following caffeine ingestion Rowing time: ↔
Christensen et al. 2014 [130]	1 female and 11 male international level rowers	6-min rowing	0.3 g/kg ingested 75 min before exercise	Caffeine: 3 mg/kg ingested 45 min before exercise	Distance covered: ↑ following isolated caffeine ingestion and combined sodium bicarbonate and caffeine ingestion Mean power: ↑ following isolated caffeine ingestion and combined sodium bicarbonate and caffeine ingestion
da Silva et al. 2018 [131]	71 male cyclists	4 × 60-s cycling at 110% of maximal power output (60-s rest) and 30-kJ time-trial test	0.3 g/kg ingested 60 min before exercise	Beta-alanine: 6.4 g/kg ingested for 28 days before exercise	Time to complete 30-kJ time-trial test: ↔
Danaher et al. 2014 [132]	8 recreationally trained males	5 × 6-s cycling (24-s rest) and cycling at 110% of the workload achieved at VO_2peak_	0.3 g/kg ingested 60 min before exercise	Beta-alanine: 4.8 g/kg ingested for 28 days followed by 6.4 g/kg ingested for 14 days before exercise	Peak power: ↔ Total work: ↔ Time to fatigue: ↑ following isolated beta-alanine ingestion and combined sodium bicarbonate and beta-alanine ingestion
de Salles Painelli et al. 2013 [133]	7 female and 7 male swimmers	100-m and 200-m swimming	0.3 g/kg ingested 60 min before exercise	Beta-alanine: 3.2 g/kg ingested for 7 days followed by 6.4 g/kg ingested for 21 days before exercise	100-m swimming time: ↓ following isolated sodium bicarbonate ingestion 200-m swimming time: ↓ following isolated sodium bicarbonate ingestion, isolated beta-alanine ingestion, and combined sodium bicarbonate and beta-alanine ingestion
Ducker et al. 2013 [134]	24 male competitive team-sport athletes	3 bout of 6 × 20-m sprints (25-s between sprints and 4-min between bouts)	0.3 g/kg ingested 60 min before exercise	Beta-alanine: 6 g/kg ingested for 28 days before exercise	Total sprint time: ↓ following isolated sodium bicarbonate ingestion First sprint time: ↓ following isolated sodium bicarbonate ingestion in bouts 2 and 3 Best sprint time: ↓ following isolated sodium bicarbonate ingestion in bouts 2 and 3
Felippe et al. 2013 [135]	10 male judo athletes	3 × SJFT (5-min rest)	0.3 g/kg ingested from 120 to 60 min before exercise	Caffeine: 6 mg/kg ingested 60 min before exercise	Number of throws bout 1: ↑ following combined sodium bicarbonate and caffeine ingestion Number of throws bout 2: ↔ Number of throws bout 3: ↑ following isolated sodium bicarbonate and combined sodium bicarbonate and caffeine ingestion Number of throws in all bouts: ↑ following combined sodium bicarbonate and caffeine ingestion
Griffen et al. 2015 [136]	9 well-trained males	6 × 10-s cycling sprints (1-min rest)	0.3 g/kg ingested in four split doses	Creatine: 20 g ingested for 7 days before exercise	Peak power: ↑ only following isolated creatine ingestion Mean power: ↑ only following combined sodium bicarbonate and creatine ingestion Relative peak power: ↑ following isolated creatine ingestion and combined sodium bicarbonate and creatine ingestion Relative mean power: ↑ only following isolated creatine ingestion Total work: ↑ only following isolated sodium bicarbonate ingestion
Higgins et al. 2016 [137]	13 non-cycling trained males	Cycling to volitional exhaustion at 100% peak mean minute power	0.3 g/kg ingested 60 min before exercise	Caffeine: 5 mg/kg ingested 60 min before exercise	Time to fatigue: ↑ following isolated caffeine ingestion and combined sodium bicarbonate and caffeine ingestion
Hobson et al. 2013 [138]	20 well-trained rowers	2000-m rowing	0.3 g/kg ingested from 240 to 120 min before exercise	Beta-alanine: 6.4 g/kg ingested for 28 days before exercise	Rowing time: ↓ following isolated beta-alanine ingestion, isolated sodium bicarbonate ingestion, and combined sodium bicarbonate and beta-alanine ingestion
Kilding et al. 2012 [139]	10 well-trained male cyclists	3-km cycling time-trial	0.3 g/kg ingested from 120 to 90 min before exercise	Caffeine: 3 mg/kg ingested 60 min before exercise	Mean power: ↑ following isolated caffeine ingestion and isolated sodium bicarbonate ingestion
Mero et al. 2004 [140]	8 female and 8 male national level swimmers	2 × 100-m swimming (10-min rest)	0.3 g/kg ingested 120 min before exercise	Creatine: 20 g ingested for 6 days before exercise	Swimming time first 100-m: ↔ Swimming time second 100-m: ↓ following combined sodium bicarbonate and creatine ingestion
Mero et al. 2013 [141]	13 competitive male swimmers	2 × 100-m swimming (12-min rest)	0.3 g/kg ingested 60 min before exercise	Beta-alanine: 4.8 g/kg ingested for 28 days before exercise	Swimming time first 100-m: ↔ Swimming time second 100-m: ↓ only following isolated sodium bicarbonate ingestion
Pruscino et al. 2008 [142]	6 elite male freestyle swimmers	2 × 200-m swimming (30-min rest)	0.3 g/kg ingested from 120 to 30 min before exercise	Caffeine: 6.2 mg/kg ingested 45 min before exercise	Swimming time first 200-m: ↔ Swimming time second 200-m: ↔
Razaei et al. 2019 [143]	8 Karatekas	Karate-specific aerobic test	0.3 g/kg ingested for 3 days and from 120 to 60 min before exercise	Caffeine: 6 mg/kg ingested 50 min before exercise	Time to fatigue: ↑ following isolated caffeine ingestion, isolated sodium bicarbonate ingestion, and combined sodium bicarbonate and caffeine ingestion
Sale et al. 2011 [144]	20 physically active males	Cycling at 110% of maximum power	0.3 g/kg ingested from 240 to 120 min before exercise	Beta-alanine: 6.4 g/kg ingested for 28 days before exercise	Total work: ↑ following isolated beta-alanine ingestion, isolated sodium bicarbonate ingestion, and combined sodium bicarbonate and beta-alanine ingestion Time to fatigue: ↑ following isolated beta-alanine ingestion, isolated sodium bicarbonate ingestion, and combined sodium bicarbonate and beta-alanine ingestion
Sarshin et al. 2021 [145]	40 taekwondo athletes	Taekwondo Anaerobic Intermittent Kick Test	0.5 g/kg ingested for 5 days before exercise	Creatine: 20 g ingested for 6 days before exercise	Peak power: ↑ in bouts 2 and 3 following combined sodium bicarbonate and creatine ingestion Mean power: ↑ bout 1 and 2 following isolated creatine ingestion, isolated sodium bicarbonate ingestion, and combined sodium bicarbonate and creatine ingestion Mean power bout 3: ↑ following isolated sodium bicarbonate ingestion, and combined sodium bicarbonate and creatine ingestion Successful kicks: ↔
Saunders et al. 2014 [146]	20 recreationally active males	3 bouts of 5 × 6-s repeated sprints (24-s rest) performed during a football specific intermittent treadmill protocol performed in hypoxia	0.3 g/kg ingested from 240 to 120 min before exercise	Beta-alanine: 6.4 g/kg ingested for 28 days before exercise and 3.2 g/kg ingested for 7 days before exercise	Mean power: ↔ Peak power: ↔
Tobias et al. 2013 [147]	37 judo and jiu-jitsu competitors	4 × 30-s modified upper body Wingate test (3-min rest)	0.5 g/kg ingested for 7 days before exercise	Beta-alanine: 6.4 g/kg ingested for 28 days before exercise	Total work: ↑ following isolated beta-alanine ingestion, isolated sodium bicarbonate ingestion, and combined sodium bicarbonate and beta-alanine ingestion; combining sodium bicarbonate and beta-alanine produced additive effects Mean power bout 1: ↑ only following combined sodium bicarbonate and beta-alanine ingestion Mean power bout 2 and 3: ↑ following isolated beta-alanine ingestion and combined sodium bicarbonate and beta-alanine ingestion Mean power bout 4: ↑ following isolated sodium bicarbonate ingestion, and combined sodium bicarbonate and beta-alanine ingestion

SJFT: Special Judo Fitness Test; ↑: significant increase; ↓: significant decrease; ↔: no significant difference

## Methods

International Society of Sports Nutrition (ISSN) position stands are invited papers on topics the ISSN editors and Research Committee identify as being of interest to our readers. Here, we briefly outline the process utilized in preparing ISSN position stands. Editors and/or the Research Committee first identify a lead author or team of authors to perform a comprehensive literature review. After the authors develop the content of the position stand, the draft is sent to leading scholars in the field for a detailed review. Following a critical review by the scholars, the paper is revised by the team of authors, approved by the Research Committee and Editors, and published as a consensus statement and the official position of the ISSN on the topic.

### History of research on sodium bicarbonate and exercise

The effects of sodium bicarbonate on exercise performance have been researched since the 1930s [148]. The first published study on this topic was carried out in the Fatigue Laboratory at Harvard University by Dennig and colleagues [148]. In this single-participant study, 10 g of sodium bicarbonate was provided before treadmill running. The authors concluded that performance was improved by establishing a pre-exercise state of alkalosis. While several studies on this topic were published in the 1950s and early 1970s [149–151], the beginning of modern-day research on sodium bicarbonate is generally linked to the study by Jones et al. [58] published in the *Journal of Applied Physiology* in 1977. In this study, 5 participants undertook 40 min of submaximal cycling prior to a cycling task to exhaustion at 95% of their maximum power on three occasions, following the ingestion of calcium carbonate (placebo condition), ammonium chloride (acidosis condition), or sodium bicarbonate (alkalosis condition). On average, participants cycled for 438 ± 120 s after ingesting sodium bicarbonate, which was significantly longer than in the acidosis (160 ± 22 s) and control (270 ± 13 s) conditions. Interestingly, sodium bicarbonate was ingested 3 h before exercise in a dose of 0.3 g/kg; this dose is used in most studies today and therefore considered a precursor to later studies. Many additional studies on sodium bicarbonate and exercise were published by this group, collectively demonstrating their major impact on the development of this field of research [61, 74, 111, 152, 153].

In the 1980s, the effects of sodium bicarbonate on exercise performance began to receive international attention, with studies published by independent research groups from Australia, Canada, France, Israel, Japan, the Netherlands, Sweden, the UK, and the USA [10, 11, 19, 42–44, 55–57, 60, 61, 65, 73–75, 89, 97, 111, 119]. Most of these studies were conducted as randomized, double-blinded, cross-over trials using small sample sizes (e.g., 6 to 7 participants per study). During this period, ergogenic effects of sodium bicarbonate were established for single and repeated high-intensity cycling, running, swimming, and muscular endurance [10, 19, 42, 44, 73, 75, 119]. Since then, there has been a large increase in the number of studies on the effects of sodium bicarbonate on exercise performance (Table 1 and Table 2), making it one of the most studied ergogenic aids.

### Mechanisms for bicarbonate absorption

Sodium bicarbonate is highly soluble in water, promptly dissociating into its constituent ions, namely sodium (Na^+^) and bicarbonate (HCO_3_^-^), on contact with aqueous solutions, including the stomach acid, as described by the following reaction:

NaHCO_3_ ➔ HCO_3_^-^ + Na^+^

HCl + HCO_3_^-^ + Na^+^ ➔ H_2_CO_3_ + Cl^-^ + Na^+^

H_2_CO_3_ ➔ CO_2_ + H_2_O

**Fig. 1:**
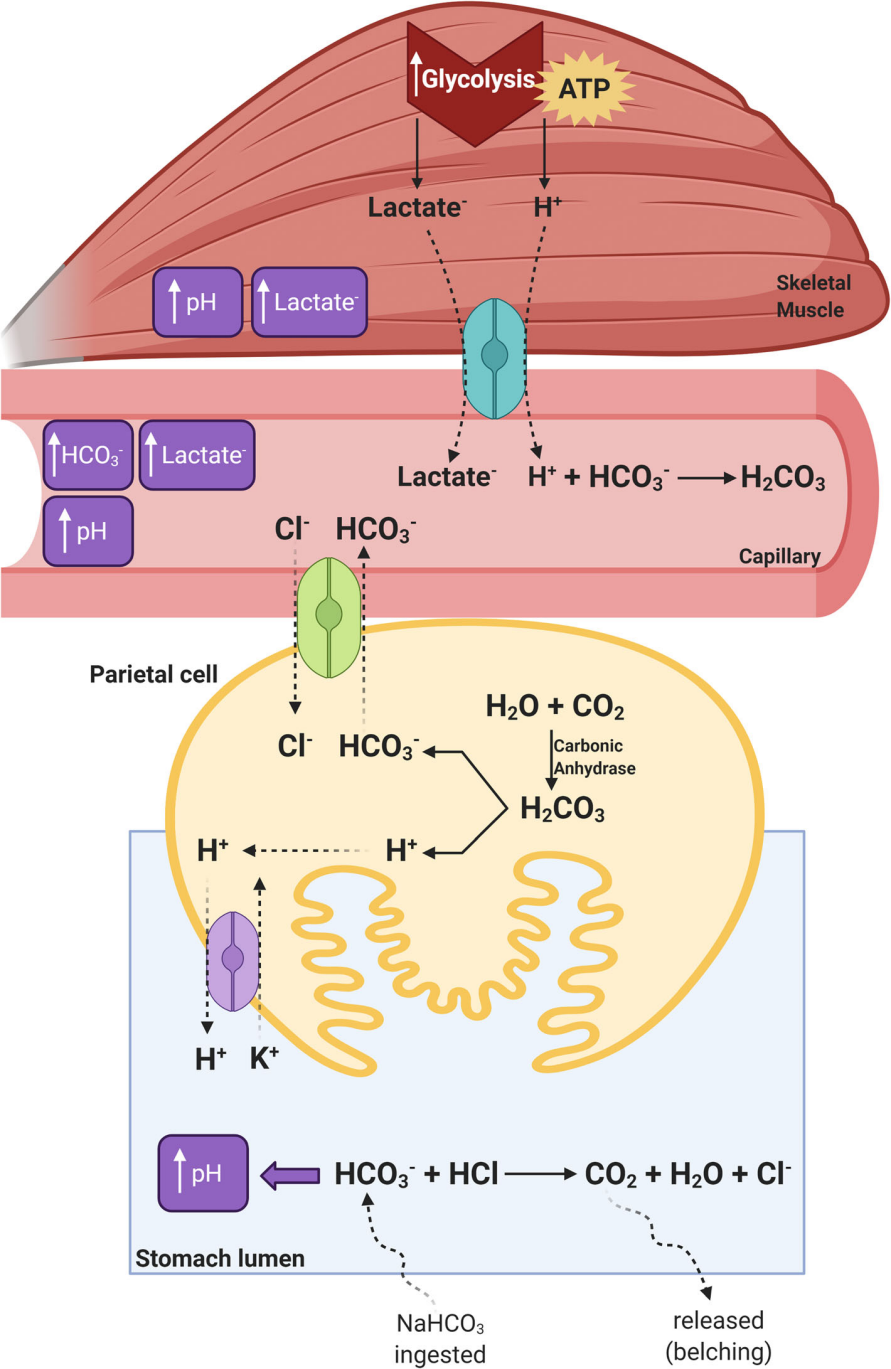
Schematic representation of the mechanism of HCO_3_^-^ absorption in the stomach and the impact of sodium bicarbonate ingestion on muscle metabolism and selected blood parameters. Sodium bicarbonate ingestion increases the concentration of HCO_3_^-^ in the stomach lumen, some of which neutralizes HCl to form CO_2_ and increases luminal pH. The rise in pH stimulates the Cl^-^/HCO_3_^-^ antiporter in the parietal cells, which transports HCO_3_^-^ into the extracellular fluid. This transport is coupled with the H-K-ATPase pump that secretes H^+^ into the stomach lumen to restore the pH. This results in increased pH and HCO_3_^-^ concentration, which increases the activity of monocarboxylate transporters (MCT1 and MCT4, represented in light blue), thereby enhancing the transport of H^+^ out of muscle cells and improving intramuscular acid-base balance. Improved pH control in the muscle cells allows higher glycolytic rates, resulting in higher rates of ATP production and higher muscle and blood lactate concentrations. Solid lines indicate reactions. Dashed lines indicate transport across membranes or movement within the cell compartment. Created using BioRender.com

Part of the ingested HCO_3_^-^ is removed via carbon dioxide (CO_2_) formation in the stomach acid. Since CO_2_ is a gas, it is released from the gastric juice on formation and then expelled. However, the rate of CO_2_ release is rather slow and increases in a concentration-dependent fashion [154]. Sodium bicarbonate ingestion increases CO_2_ formation, thus increasing the requirement for and rate of CO_2_ release, a mechanism that explains commonly reported side-effects of sodium bicarbonate such as belching and bloating. Despite the removal of some HCO_3_^-^ due to acid neutralization in the stomach, the alkalization of the gastric juice following sodium bicarbonate ingestion will likely stimulate the basolateral Cl^-^/HCO_3_^-^ antiporter in parietal cells mediated by apical gastric H^+^/K^+^-ATPase, leading to increased HCO_3_^-^ transport into the blood [155, 156] (Fig. 1).

In addition to HCO_3_^-^ absorption in the stomach, other absorption mechanisms exist in the human intestine [157]. Plausibly, the high doses of sodium bicarbonate commonly ingested (see section “Sodium bicarbonate dose”) do not entirely react with the stomach acid since the bicarbonate load exceeds the amount of acid in the stomach. Thus, some HCO_3_^-^ will enter the intestine and reach the jejunum, where it can be absorbed. This mechanism has been shown to be concentration-dependent and to involve either coupled Na^+^ absorption or active H^+^ secretion [157]. These multiple mechanisms seem to account for the rapid increase in plasma HCO_3_^-^ concentration that occurs following acute sodium bicarbonate ingestion [19].

### Mechanisms for the ergogenic effect of sodium bicarbonate

**Fig. 2:**
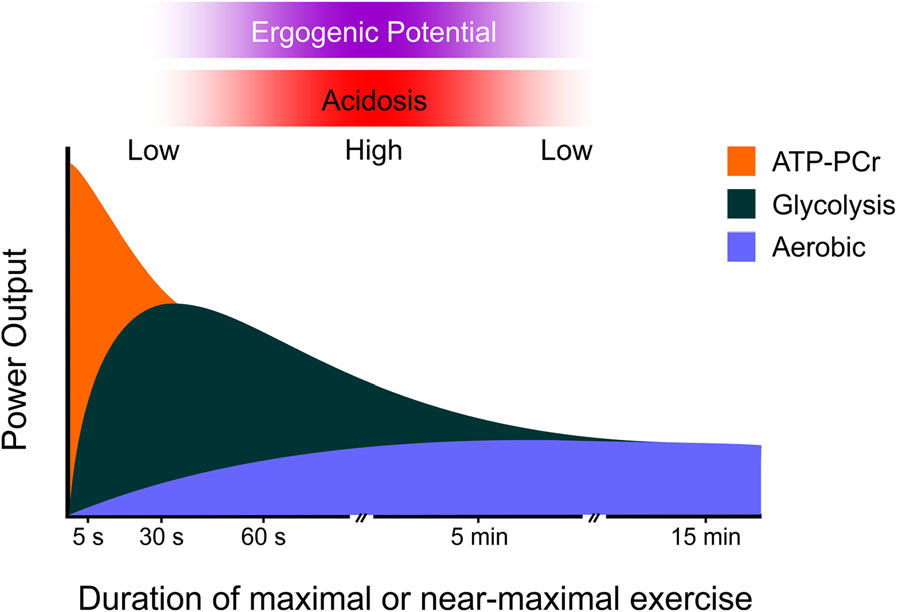
Illustration of the contribution of different energy systems to the production of ATP to sustain maximal or near-maximal exercise over a given amount of time. The horizontal gradient-filled bars indicate exercise intensity/duration zones that are more (filled) or less (shaded) likely to result in acidosis and thus benefit from sodium bicarbonate supplementation

During high-intensity short-term exercise, the rate of intramuscular ATP hydrolysis exceeds the maximum rate of ATP re-synthesis by mitochondria. Therefore, ATP production heavily relies on anaerobic systems, namely phosphoryl-creatine (ATP-PCr) hydrolysis and glycolysis. The contribution of each of these systems to ATP production varies according to different factors, such as age [158] and training [159]. However, exercise intensity is a major determinant of the energy system contribution [160]. Illustrated in Fig. 2, the higher the intensity, the higher the predominance of the ATP-PCr system. As intensity reduces, the contribution of ATP-PCr reduces accordingly and the ATP demands are increasingly met by glycolysis [160]. Thus, the metabolic perturbations elicited by maximal all-out exercises lasting less than 30 s are distinct from those that occur during intense exercise lasting from ~30 s to ~5 min. While the former is characterized by rapid phosphoryl-creatine depletion, the latter is characterized by substantive accumulation of lactate and H^+^ in both intra and extracellular fluids. The differences in the metabolic perturbations induced by exercise of different intensities and durations seem to be related to the ergogenic potential of sodium bicarbonate supplementation. Specifically, a large body of evidence indicates that exercise that is more reliant on glycolysis and thus results in greater H^+^ accumulation is more likely to benefit from sodium bicarbonate supplementation in comparison to exercise that is too short or too long to result in marked acidosis [1].

In response to intense contractile activity, muscle temporarily loses much of its ability to generate force and power in a process defined as fatigue. The associated accumulation of metabolites and ions is thought to play a causative role in the fatigue process, although the exact causes of fatigue continue to be debated [161–163]. Accumulation of H^+^ (i.e., muscle acidosis or a decrease in muscle pH) has been shown to be an important contributing factor to fatigue due to its inhibitory effects on key glycolytic enzymes [164], depressing effects on Ca^2+^ sensitivity, and direct effects on cross-bridge cycling [165].

High-intensity exercise is also associated with marked changes in the intracellular and extracellular concentrations of various ions [163, 166]. One important alteration is the release of K^+^ from the muscle cells to the interstitium, leading to increased interstitial K^+^ concentration. Whilst moderate elevations in extracellular K^+^ may enhance muscle function [167], very high interstitial K^+^ can depolarize sarcolemma and T-tubule membranes, thus affecting muscle fiber conduction [168] and excitability [169], ultimately leading to muscle fatigue. Exercise also imposes important disturbances to Na^+^, Cl^-^ and Ca^2+^ transmembrane gradients, which also contribute to the loss of excitability [162]. Reduced sarcolemmal and T-tubule excitability are linked with decreased Ca^2+^ release from the sarcoplasmic reticulum, which is another important contributor to fatigue [152, 166, 170]. During fatigue, the rise in Mg^2+^ and inorganic phosphate concentrations, as well as the fall in ATP concentrations, in skeletal muscle have also been linked with reduced force production and, therefore, appear to contribute to fatigue.

The ergogenic mechanisms of sodium bicarbonate are not yet fully understood. Nevertheless, an increase in extracellular buffering capacity is a widely accepted mechanism for the ergogenic effect of sodium bicarbonate. Since the sarcolemma is not permeable to HCO_3_^-^, sodium bicarbonate ingestion leads to an increase in plasma HCO_3_^-^ concentration in a dose-dependent manner [19, 33, 77]. This is associated with changes in blood acid-base balance, including an increased pH and base excess, which characterize a state of metabolic alkalosis.

Increased extracellular pH leads to a greater transmembrane H^+^ concentration gradient [153] that stimulates H^+^ and lactate co-transport out of exercising muscle cells, most likely via monocarboxylate transporters MCT4 and MCT1. Evidence from *in vitro* studies also suggests that increased extracellular HCO_3_^-^ concentration may contribute to lactate efflux from skeletal muscle [171]. Direct evidence of increased lactate efflux following sodium bicarbonate ingestion has been provided by Hollidge-Horvat et al. [153]. Given that lactate transport is stoichiometrically coupled with H^+^, increased lactate efflux also indicates increased H^+^ efflux during exercise, therefore reducing the intramuscular H^+^ accumulation. Following sodium bicarbonate ingestion, some studies reported higher intramuscular pH during exercise [19, 110], although conflicting data exist [35]. A study measuring pH with phosphorous magnetic resonance spectroscopy during exercise confirmed a delayed onset of intramuscular acidosis with sodium bicarbonate ingestion [96], providing support for the notion of increased H^+^ efflux and improved intramuscular acid-base balance as a primary ergogenic mechanism following sodium bicarbonate supplementation.

Improved control of intramuscular pH during exercise results in increased glycolytic rates and higher rates of ATP re-synthesis to sustain higher exercise demands. Indeed, studies with muscle biopsies have shown increases in post-exercise muscle lactate content following sodium bicarbonate ingestion, as well as increased glycolytic activity and glycogen utilization [7, 10, 111, 161]. More direct evidence of increased glycolytic rates has been provided by studies showing increased rates of glycogen utilization and lactate production during exercise after sodium bicarbonate ingestion [153]. Recent studies assessing the whole-body contribution of the energy systems during exercise after sodium bicarbonate ingestion [68, 131] have also reported an increased glycolytic contribution to explain improvements in high-intensity performance via longer periods in which higher rates of ATP re-synthesis are sustained.

In addition to the improved glycolytic metabolism in the exercising muscle cells, improved pH regulation stemming from sodium bicarbonate supplementation may also have a direct effect on the cross-bridge cycle, potentially attenuating the suppressive effects of acidosis on muscle contractility [172]. Moreover, because pH alters Ca^2+^ sensitivity [173], improved intramuscular pH regulation might also result in increased force production in response to similar cytosolic Ca^2+^ concentration during muscle contraction. Whether sodium bicarbonate ingestion can indeed affect the cross-bridge cycle and Ca^2+^ sensitivity in human muscle remains to be experimentally determined.

The traditional Henderson’s interpretation of acid-base regulation has been expanded by the physicochemical approach proposed by Stewart in the 1980s [174, 175]. This interpretation based acid-base as disturbances, having either respiratory or metabolic origins, to describe three independent variables that collectively drive H^+^ and HCO_3_^-^ ion concentrations as dependent variables. The independent variables are: the Strong Ion Difference ([SID]), defined as the excess of strong cations in relation to strong anions; PCO_2_; and the total concentration of weak acids and bases ([A_TOT_]). It is assumed that any changes in plasma Na^+^, Cl^-^, K^+^ or Lac^-^ concentrations, as well as in PCO_2_ with sodium bicarbonate ingestion may directly affect plasma pH and plasma HCO_3_^-^ concentrations [109, 152]. Changes in muscle intracellular and extracellular strong ion concentrations can therefore affect not only acid-base status but can also directly modulate sarcolemmal and transverse membrane excitability. Alkalosis induced by sodium citrate ingestion has been shown to attenuate the increase in interstitial K^+^ concentration during exercise [176], thereby minimizing an important factor contributing to fatigue. A study with sodium bicarbonate ingestion [109] showed lower K^+^ concentration and an increased K^+^ efflux from the exercising muscle, along with an increase in post-exercise K^+^ reuptake, indicating enhanced Na^+^,K^+^-ATPase activity. Thus, sodium bicarbonate helps to minimize K^+^ disturbances in muscle and potentially also preserve sarcolemmal excitability. Intense exercise also induces disturbances in Na^+^, Cl^-^, and Ca^2+^ transmembrane gradients; altogether, these changes lead to transsarcolemmal and T-tubular depolarization and loss of excitability [162], a condition that results in impaired force production and can be mitigated by sodium bicarbonate ingestion [109].

### Effects of sodium bicarbonate on high-intensity exercise performance

A number of studies have investigated the effects of sodium bicarbonate on high-intensity exercise performance. Many, but not all, studies have reported an ergogenic effect of sodium bicarbonate. However, the duration of the exercise task is an important component to consider when interpreting the evidence.

#### Single-bout running and cycling

The effects of sodium bicarbonate on high-intensity, single-bout running or cycling have been thoroughly explored [6, 10, 27, 28, 38, 40, 44–47, 49, 50, 52, 55–57, 70, 76–79, 90, 98, 111, 114, 115, 119]. A recent meta-analysis pooled results from studies exploring the effects of sodium bicarbonate on performance in the Wingate test, which in its original form involves 30-s all-out cycling [177]. In this meta-analysis, no significant difference was found between the effects of a placebo and sodium bicarbonate on mean or peak power [177]. The pooled effect size and corresponding 95% confidence intervals (CI) for mean power were in the range of trivial/very small effects (Cohen’s *d*: 0.02; 95% CI: –0.07, 0.11), suggesting any actual effect of sodium bicarbonate on this outcome would likely be negligible from a practical perspective, at least for most individuals.^1^ Therefore, it seems that a single-bout, high-intensity task lasting 30 s may not be of sufficient duration to benefit from sodium bicarbonate.

The minimum duration of a high-intensity task to experience an ergogenic effect of sodium bicarbonate is yet to be established. However, McNaughton [78] examined the effects of sodium bicarbonate supplementation on performance in cycling tasks lasting 10, 30, 120, and 240 s. Whereas no significant differences in total work and peak power were found between sodium bicarbonate and placebo trials for cycling tests lasting 10 or 30 s, significant performance improvements were shown for the two cycling tests of longer duration (120 and 240 s). Running-based investigations have focused on distances ranging from 400 m to 1500 m, lasting from 57 to 254 s. For example, Wilkes et al. [119] included 6 male university runners who performed an 800-m run following the ingestion of a placebo or 0.3 g/kg of sodium bicarbonate. In this study, running time following placebo ingestion was 125.1 ± 4.9 s and consumption of sodium bicarbonate reduced the time needed to complete the test on average by 2.9 s (1.8%). Other studies reported an average reduction in the time required to complete 400-m and 1500-m runs of 1.5 s and 3 s (1.1% to 2.9%) [6, 44]. In addition to reducing the time to finish a run, sodium bicarbonate supplementation has also been shown to delay fatigue during high-intensity running. Van Montfoort et al. [114] evaluated running on a treadmill using an incline of 2% and speeds between 19 and 23 km/h, set to elicit maximum effort in 1 to 2 min. The ingestion of sodium bicarbonate was ergogenic, as the average running time was 77.4 s and 82.3 s (6%) following placebo and sodium bicarbonate ingestion, respectively. Overall, it seems that the minimum duration of a single-bout, high-intensity task to experience an ergogenic benefit with sodium bicarbonate needs to be longer than 30 s.

In terms of the upper limits of the task duration, several studies [45–47, 49, 50, 52, 53, 86] have used high-intensity cycling or running tests lasting between 4 and 12 min and reported ergogenic effects of sodium bicarbonate ingestion. For example, Gough et al. [47, 48] used 4-km cycling time trials (lasting around 6 min) and observed that sodium bicarbonate reduced the time to complete this test by 5 – 8 s (~2%). Mueller et al. [86] used a test involving cycling to exhaustion at critical power that lasted on average 12 min and documented an ergogenic effect of sodium bicarbonate ingestion. Overall, the evidence indicates that sodium bicarbonate may improve single-bout high-intensity cycling and running if the exercise test is of sufficient intensity and lasts approximately between 30 s and 12 min. Single-bout exercise tasks of longer duration, and thus lower intensity, are less likely to be influenced by sodium bicarbonate ingestion.

Stephens and colleagues examined the effects of sodium bicarbonate on 60-min cycling performance [110]. Even though the ingestion of 0.3 g/kg of sodium bicarbonate significantly increased plasma bicarbonate concentration and decreased plasma and muscle H^+^, exercise performance was not affected [110]. Three other studies also did not find a significant difference between the effects of placebo and sodium bicarbonate in different exercise tasks lasting from 20 to 68 min (i.e., running to exhaustion, cycling to exhaustion, and 40-km cycling time trial) [39, 61, 87]. However, three studies reported a positive effect of sodium bicarbonate on performance in exercise tasks of similar duration [36, 43, 80]. McNaughton et al. [80] found that sodium bicarbonate ingestion increased mean power during a 1-h cycling exercise, while others reported that time to fatigue in cycling or running to exhaustion (lasting from 26 to 50 min) was delayed following sodium bicarbonate ingestion [36, 43]. While this area requires further research, it should be mentioned that sodium bicarbonate may be beneficial during prolonged exercise, when such exercise involves periods of increased intensity. For example, athletes competing in road cycling or track running commonly finish the race with an all-out sprint, which may be enhanced with sodium bicarbonate supplementation [178]. A recent study provided sodium bicarbonate in the dose of 0.3 g/kg, supplemented before and during a 3-h simulated cycling race that involved a 90-s all-out sprint at the end of the race [21]. Sodium bicarbonate ingestion enhanced mean power in the 90-s sprint by ~3% [21]. Based on this recent evidence, it seems that sodium bicarbonate may enhance performance during endurance events if they include sprints during or at the end of the competition.

#### Repeated-bout running and cycling

While sodium bicarbonate ingestion appears ergogenic for single-bout running and cycling tasks, its effects are proposed to be more pronounced in multiple bouts of maximal exercise since the performance in the latter bouts might be more affected by acidosis [19]. Costill et al. [19] used a protocol consisting of 4 × 1-min cycling (including 1-min rest periods), with the fifth sprint performed to exhaustion. This final sprint was extended by an average of 47 s following sodium bicarbonate ingestion (placebo: 113 ± 41 s; sodium bicarbonate: 160 ± 63 s), demonstrating an increase in exercise performance during high-intensity, repeated-bout exercise.

While sodium bicarbonate ingestion does not seem to be ergogenic for a single 30-s all-out exercise bout, several studies have investigated the effects of sodium bicarbonate ingestion on repeated maximal 30-s efforts [4, 85, 88, 89, 122, 123, 125]. For example, Artioli et al. [4] used a 4 × 30-s arm cranking test with intervening 3-min rest and observed that sodium bicarbonate ingestion increased peak and mean power only in bouts 3 and 4. These results were confirmed by another study from the same research group [88]. However, other studies have used similar protocols and did not show ergogenic effects of sodium bicarbonate [122, 123]. The lack of an effect in these studies might be due to the longer duration of rest intervals between cycling tasks (15 to 30 min) [122, 123]. The benefits of sodium bicarbonate may be greatest when employing rest intervals of shorter duration [60]. Indeed, this is supported by a recent meta-analysis [177] that included 10 studies and found an ergogenic effect of sodium bicarbonate on mean power in bout 2 of maximal 30-s cycling (pooled Cohen’s *d* = 0.09) and bout 4 (pooled Cohen’s *d* = 0.62). In bout 3, a significant effect of sodium bicarbonate was found on mean power (pooled Cohen’s *d* = 0.40), albeit only when considering studies that used shorter rest intervals between repeated tests (i.e., 3 to 6 min) [177]. Similarly, Siegler et al. [101] reported that sodium bicarbonate supplementation increased average speed in a protocol involving three bouts of running at maximum speed for 30 s followed by a 3-min rest, but only in the third bout [101]. As a result, total distance covered was also greater in the sodium bicarbonate trial.

Subsequent research has observed an ergogenic effect of sodium bicarbonate on multiple bouts of cycling and running of even shorter duration. Bishop et al. [7] used a repeated-sprint protocol where the participants were required to perform 5 × 6-s cycle sprints with intervening 30-s rest periods, with the analyzed outcomes being total work and peak power. Total work across all sprints was higher following the ingestion of sodium bicarbonate (0.4 g/kg), and this was largely attributed to the increase in peak power in sprints 3, 4, and 5. In other words, sodium bicarbonate enhanced performance by attenuating the fatigue-induced decline in power during repeated-sprints. Lavender and Bird [65] evaluated performance following sodium bicarbonate ingestion in a protocol involving 10 × 10-s cycle sprints, interspersed with 50-s rest periods. Sodium bicarbonate ingestion improved mean power in 8 sprints (sprints 2 and 3, and 5 to 10) and peak power in 2 out of the 10 performed sprints (sprints 2 and 10). In both of these studies, performance in the first sprint was not affected by sodium bicarbonate ingestion, further demonstrating no benefit of this supplement on shorter duration exercise tasks [7, 65].

The Yo-Yo intermittent recovery test [179] is widely used to assess performance in interval running, which is particularly relevant for many team sports. It determines an individual’s capacity to undertake, repeatedly perform, and recover from high-intensity running. In this test, the individual must run 2 × 20-m distances at progressively increasing speeds, interspersed with a 10-s rest interval [179]. This test has two types, “level 1” and “level 2”, with the latter starting at a higher speed and requiring a greater contribution from anaerobic energy systems [179]. Due to its popularity in practice, studies have also explored the effects of sodium bicarbonate on performance in this test [25, 63, 69, 113]. For example, Marriot et al. [69] included 12 male team-sport athletes who ingested 0.4 g/kg of sodium bicarbonate or placebo 60 to 90 min before exercise. Sodium bicarbonate increased the distance covered in the level 2 Yo-Yo intermittent recovery test by 23% [69]. A recent meta-analysis also reported an ergogenic effect of sodium bicarbonate on performance in this test (pooled Cohen’s *d* = 0.36; 16%) [180]. As the Yo-Yo test has been reported to correlate with some sport-specific outcomes (e.g., amount of high-intensity running performed at the end of each half of a game) [179], athletes competing in intermittent sports, such as basketball, football, hockey, and rugby, may consider supplementation with sodium bicarbonate to acutely improve their performance. At least theoretically, the effects of sodium bicarbonate are likely to be greater in the level 2 versions of the Yo-Yo test, which require a larger contribution from the anaerobic energy system [179]. Still, future research is needed to directly compare the effects of sodium bicarbonate on level 1 and level 2 Yo-Yo test performance.

#### Single-bout rowing

At the Olympic Games and world championships, all rowing races are performed over a 2000-m distance. It is estimated that approximately 30% of the energy necessary to complete a 2000-m row is derived from anaerobic sources [181], meaning that sodium bicarbonate may be beneficial in such exercise tasks.

Several studies have investigated the effects of sodium bicarbonate on rowing using a 2000-m rowing ergometer time trial as a measure of performance [15, 54, 129, 138]. Carr et al. [15, 129] conducted two studies to explore the effects of sodium bicarbonate on performance in the 2000-m rowing ergometer test among well-trained rowers. They did not find an ergogenic effect of sodium bicarbonate on rowing performance using either a single-dose (0.3 g/kg consumed 120 to 90 min before the test) or multiple-day (0.5 g/kg per day consumed for 3 days before the test) supplementation protocol. In their study that used the single-dose protocol, average rowing time following placebo and sodium bicarbonate ingestion was 403.8 ± 23.4 s and 404.4 ± 23.4 s, respectively. However, limitations of these studies are a small sample including only 7 to 8 participants and high intra-individual variability in performance, blood alkalosis, and gastrointestinal symptoms [15, 129]. In a much larger sample (*n* = 20 club-level rowers), sodium bicarbonate ingestion in the dose of 0.3 g/kg consumed from 240 to 120 min before exercise improved 2000-m rowing performance [54]. In this study, the time needed to complete 2000 m of rowing was 410.7 ± 14.9 s following sodium bicarbonate ingestion and 412.0 ± 15.1 s following placebo ingestion. This study also analyzed the 500-m split times and reported a reduction in rowing time in the second half of the race (i.e., from 1000 m to 2000 m), supporting previous findings that sodium bicarbonate is effective towards the latter stages of exercise.

The positive effects of sodium bicarbonate on rowing performance were also established in a recent meta-analysis [182] that pooled the results of all these previous studies. This analysis reported an improvement in rowing performance by 1.4% (90% CI: 0.1%, 2.6%) following the ingestion of sodium bicarbonate compared to placebo. While such an improvement may seem small, it is likely to be practically important for competitive rowing, where small differences often determine placings. For example, at the 2016 Rio Olympic Games, the first and second places in the men’s single scull event were determined by a photo-finish. It is estimated that improvements in performance as small as 0.3% may have meaningful effects on performance outcomes in rowing competitions, highlighting that rowers may consider supplementation with sodium bicarbonate [183]. Overall, we conclude that sodium bicarbonate is likely to have a small but positive effect on rowing performance.

#### Single-bout swimming

Competitive swimming is a single-bout event (e.g., 100-m swimming, 200-m swimming). Therefore, studies that examined the effects of sodium bicarbonate on single-bout swimming tasks likely offer the most practically important findings for competition [59, 64, 67, 92, 121, 142]. Lindh et al. [67] conducted a study that involved nine elite male swimmers who performed 200-m freestyle swimming after the ingestion of placebo or 0.3 g/kg of sodium bicarbonate. In this study, swimming time following placebo ingestion was 114 ± 3.6 s. Ingestion of sodium bicarbonate reduced the time needed to complete 200-m of swimming on average by 1.8 s (1.6%). Despite this finding, other studies investigating the effects of sodium bicarbonate on 200-m swimming performance and did not report significant ergogenic effects of sodium bicarbonate among highly trained swimmers [59, 142]. Reasons for this discrepancy in the findings are currently unclear, but they may be associated with the individual variation in responses to sodium bicarbonate supplementation and/or due to the small samples (*n* = 6 to 7) included in these studies [59, 142].

To address the limitation of small sample sizes commonly observed in the literature, one meta-analysis explored the effects of sodium bicarbonate on swimming performance [184]. Sodium bicarbonate was not found to be ergogenic for 100-yard (91.4-m) and 100-m swimming tests lasting between 50 and 60 s. Nevertheless, an ergogenic effect was shown in a subgroup analysis that included only 200-m and 400-m swimming tests, lasting between 112 s and 270 s [184]. Based on this analysis and consistent with the results for running and cycling, it seems that sodium bicarbonate may improve swimming performance in longer-distance events. Even though the magnitude of improvement was small (pooled Cohen’s *d* = 0.22; 1.3%), it may be of practical importance as placings in swimming competitions are often determined by narrow margins. For example, at the 2016 Olympic Games finales in the 200-m butterfly stroke, the difference between first and second place was only 0.04 s (i.e., 1:53.36 vs. 1:53.40-min). Data suggest that improvements in swimming performance of ~0.4% may represent a practically relevant effect in regard to event outcomes [185]; therefore, sodium bicarbonate supplementation seems to be beneficial in swimming competitions.

#### Repeated-bout swimming

Several studies have also explored the effects of sodium bicarbonate on interval swimming performance, which is relevant for the interval-based training practices of swimmers [42, 102, 124, 186]. In the first study on this topic, Gao et al. [42] examined the effects of sodium bicarbonate on swimming velocity using a 5 × 100-yard swimming protocol, with a 2-min rest between intervals. Results showed that sodium bicarbonate increased swimming velocity in swimming intervals 4 and 5 (~2%). A similar ergogenic effect (1.1% to 2%) was also reported in studies using 8 × 25-m (5-s rest) and 4 × 50-m swimming (1-min rest) protocols [102, 124].

Two studies that used interval swimming protocols did not report an ergogenic effect of sodium bicarbonate [14, 112]. One of them [14] used a protocol involving 6 × 100-m swimming, but the rest interval between bouts lasted for 6 min, much longer than in the studies that reported ergogenic effects (with rest periods between 5-s and 2-min). The other study [112] employed a 56 × 10-m sprint swimming protocol (~7 s per sprint), where the rest interval ranged between 17 s and 5 min. The study’s lack of a significant ergogenic effect may be because the outcome variable was the average time across all 56 sprints. This is a limitation, given that the effects of sodium bicarbonate may not have been uniform in all the sprints. In the early work by Gao et al. [42], the ergogenic effects of sodium bicarbonate on 100-yard swimming performance were only recorded in bouts 4 and 5. Therefore, future studies that use interval swimming should consider analyzing the effects of sodium bicarbonate on each sprint separately. Overall, it seems that sodium bicarbonate may enhance interval swimming performance and that these effects are more pronounced when using shorter rest intervals between swimming bouts and longer swimming distances.

### Effects of sodium bicarbonate on performance in combat sports

Competitive events in many combat sports consist of high-intensity actions of short duration that are interspaced with brief intervals of lower exertion [187, 188]. For example, an analysis of movements in judo showed that most actions were of high intensity, lasted between 20 and 30 s, and were separated by short periods of lower exertion lasting from 5 to 10 s [187]. Due to their structure, many combat sports (e.g., judo, wrestling) rely heavily on glycolysis [187], which explains the interest of researchers to explore the effects of sodium bicarbonate supplementation on performance exercise tasks relevant to these sports.

Three studies involving judo athletes [4, 135, 147] have examined the effects of sodium bicarbonate on the number of throws in three bouts of the “Special Judo Fitness Test”. The “Special Judo Fitness Test” involves three periods lasting between 15 s and 30 s during which the participant attempts to complete as many throws as possible on two other individuals. Supplementation with sodium bicarbonate increased the number of throws by ~2 (6%) in the “Special Judo Fitness Test” bouts 2 and 3 [4, 135].

Similar studies have also been conducted with athletes competing in boxing, karate, taekwondo, and wrestling [32, 34, 68, 100, 143]. In a study including amateur boxers [100], 0.3 g/kg of sodium bicarbonate ingested 60 min before exercise increased the number of punches performed during four rounds of sparring, where each round lasted 3 min with a 1-min rest interval. Sodium bicarbonate ingestion also prolonged the time to fatigue during a Karate-specific aerobic test in 8 karate athletes, and it increased attack time during simulated taekwondo combat [68, 143]. Durkalec-Michalski et al. [32] examined the effects of sodium bicarbonate ingestion on the number of throws during a dummy throw test in a cohort of elite competitive wrestlers. Results did not show an ergogenic effect of sodium bicarbonate (*p* = 0.07), using a multiple-day, progressive protocol of sodium bicarbonate supplementation (from 0.025 g/kg per day to 0.100 g/kg per day over 10 days). However, a follow-up study by the same research group [34] reported an increase in the number of throws (~2) in the same dummy throw test using the same supplementation protocol, even though the improvement was found only in male participants. Overall, most studies suggest that sodium bicarbonate is an effective supplement for enhancing performance in combat sports, such as boxing, judo, karate, taekwondo, and wrestling. Therefore, athletes competing in combat sports may consider using supplementation with sodium bicarbonate to improve their performance. However, future research is needed to explore the effects of sodium bicarbonate supplementation specifically in combat sport competitions.

### Effects of sodium bicarbonate on resistance exercise performance

A considerable amount of evidence exists on the effects of sodium bicarbonate supplementation on resistance exercise performance, particularly for two outcomes, muscular endurance and muscular strength [3, 16, 18, 30, 37, 71, 73, 104–108, 118]. Muscular endurance is commonly assessed as the maximum number of completed repetitions of a movement with a given load or as the maximum duration of maintaining isometric force production. A recent meta-analysis [189] of 12 studies showed sodium bicarbonate supplementation to be ergogenic for muscular endurance (Cohen’s *d* = 0.37; 95% CI: 0.15, 0.59). While an acute improvement in muscular endurance might be expected following sodium bicarbonate intake, its magnitude will likely depend on a number of factors.

Two particularly important factors to consider are the external load and duration of the task. Maughan et al. [73] evaluated the effects of consuming 0.3 g/kg of sodium bicarbonate on time to maintain an isometric contraction at 80%, 50%, and 20% of maximum isometric strength. In this study, an ergogenic effect was observed when utilizing the lowest load, increasing the time of the isometric contraction from 210 ± 77 s in the placebo trial to 227 ± 65 s after sodium bicarbonate ingestion (8%). With the two higher loads, the participants could only maintain the contraction from 20 to 58 s. The duration of these tasks might have been too short to observe a benefit from sodium bicarbonate supplementation. Alternatively, the restricted blood flow to the contracting muscles with these more intense isometric contractions may have minimized lactate, H^+^, and K^+^ release from muscles and thus diminished benefits of sodium bicarbonate supplementation. Another study [18] used isokinetic knee flexion and extension test that lasted 85 s, where total work was used as a proxy of muscular endurance and found an ergogenic effect of sodium bicarbonate. Similar to the findings observed for high-intensity exercise, the evidence is suggesting that the duration of the muscular endurance task may need to exceed ~1 min for sodium bicarbonate to be ergogenic, at least when a single set is performed.

Another factor that needs to be considered is the set protocol. Webster et al. [118] used a protocol where the participants first performed 4 sets of 12 repetitions of the leg press, followed by one set performed to muscular failure at 70% of one-repetition maximum (1RM) following sodium bicarbonate or placebo ingestion. The outcome variable in this study was the number of completed repetitions in the fifth set. The study did not find significant differences in the number of repetitions between the two conditions (average of 18 and 19 repetitions for placebo and sodium bicarbonate, respectively). Assuming that this study adopted a tempo of 1 s for the eccentric and concentric phase of the movement, the duration of the exercise task would only have been around 40 s, which might explain the absence of an ergogenic effect. However, when multiple set protocols performed to muscular failure were used, an ergogenic effect of sodium bicarbonate on muscular endurance was reported [16, 30]. For example, Carr et al. [16] assessed the number of repetitions performed in 13 sets across three lower-body exercises. An ergogenic effect was observed, as the total number of performed repetitions was increased from 157 ± 15 to 164 ± 15 (4.5%) in the placebo and sodium bicarbonate trials, respectively. This study only analyzed the total number of repetitions across all exercises and did not evaluate performance in each set separately. It is conceivable that the effects of sodium bicarbonate increase with increasing the number of sets. This hypothesis is somewhat supported by the work of Duncan et al. [30]. After the ingestion of sodium bicarbonate and placebo, the participants in their study were able to complete, on average, 12 repetitions in the first set of squats at 80% of 1RM. In the second set that took place after a 3-min rest interval, the participants performed 11 repetitions in the sodium bicarbonate trial and 7 repetitions in the placebo trial. Favorable effects of sodium bicarbonate were also observed in the third set (9 vs. 6 repetitions). Therefore, it seems that the effects of sodium bicarbonate on muscular performance are more pronounced when using multiple-set protocols.

Muscular strength, commonly evaluated using isometric, isokinetic, or isotonic tests, is another important muscular quality in resistance exercise [190]. Given that muscular strength tests are characterized by very brief duration and maximal exertion, it appears less likely that sodium bicarbonate would be ergogenic for this outcome. Indeed, a recent meta-analysis [189] did not find a significant difference between the effects of sodium bicarbonate and placebo on muscular strength (Cohen’s *d* = –0.03; 95% CI: –0.18, 0.12). Furthermore, the upper and lower limits of the 95% CI were in the range of trivial/very small effects, suggesting that even if there is a true effect of sodium bicarbonate on muscular strength in the population, it is likely practically negligible. While sodium bicarbonate may not increase strength *per se*, it might prevent the fatigue-induced decline in strength [3]. Ansdell et al. [3] evaluated the effect of sodium bicarbonate supplementation on isometric strength of the knee extensors assessed before a basketball game and after each quarter. The authors observed that the decline in strength was on average 9% when sodium bicarbonate was ingested, compared to a 15% decline in the placebo condition [3]. Although sodium bicarbonate may not enhance muscle strength directly, this supplement seems to enhance the capacity to train maximally, thus leading to increased strength gains with resistance training. Indeed, attenuating the decline in strength during multiple sets has been found to contribute to greater gains in strength [191].

In animal models, studies commonly report that increases in bicarbonate concentration and/or intracellular accumulation of H^+^ do not impact maximum force production, even though these effects may be temperature-dependent [192, 193]. This lack of an effect on force production might also explain why sodium bicarbonate supplementation in humans generally does not enhance maximum strength. However, data using animal models also show that a decline in pH negatively affects muscle conduction velocity, a metric that is positively related to the rate of force development (RFD) [194, 195]. Given that one of the mechanisms of sodium bicarbonate is buffering of H^+^, the ingestion of this supplement may positively impact RFD. A series of studies explored the effects of sodium bicarbonate on RFD following either 30 s of cycling, maximum strength testing, or five sets of knee extensions [104, 106–108]. RFD was higher following sodium bicarbonate ingestion in three of these studies. These findings might be of practical importance as RFD is associated with several aspects of athletic performance, such as sprinting and jumping [196, 197]. This might partially explain some of the positive results shown for the effect of sodium bicarbonate supplementation on repeated-sprint activities. While these initial findings are promising, future work is still needed, as only a few studies have focused on this outcome.

### Effects of sodium bicarbonate on training adaptations

Most studies in the field have explored the acute effects of sodium bicarbonate supplementation. However, a handful of studies have also explored the effect of long-term supplementation with sodium bicarbonate. Edge et al. [35] used a volume- and intensity-equated cycling interval training program in which female student participants (*n* = 16) were randomized to consume 0.4 g/kg of sodium bicarbonate one hour before every training session for 8 weeks (overall 24 sessions) or to ingest the same amount of placebo. After 8 weeks of training, greater improvements in the lactate threshold (26% vs. 15%) and time to fatigue while cycling at 100% of peak oxygen uptake (164% vs. 123%) were shown in the group ingesting sodium bicarbonate. It is important to emphasize that the capacity tests were performed without prior acute supplementation, meaning these differences can be attributed to greater adaptations throughout the training period with sodium bicarbonate supplementation. The authors suggested that less disturbance to metabolic homeostasis associated with the use of sodium bicarbonate around exercise sessions might have promoted muscle protein balance, contributing to greater increases in intracellular muscle proteins involved in mitochondrial respiration or ion balance [35]. As suggested in a subsequent study conducted with rats, the effects might indeed be attributed to greater mitochondrial adaptations in the group ingesting sodium bicarbonate [198].

Another study [117] explored the effect of high-intensity interval training coupled with sodium bicarbonate supplementation in a group of 20 recreationally active men. Following six weeks of high-intensity interval training, incorporating 18 training sessions, the group ingesting sodium bicarbonate prior to every training session experienced greater improvements in relative peak power during 30-s all-out cycling than the group ingesting a placebo (21% vs. 10%). However, these findings were not confirmed in two other studies [29, 108]. Sodium bicarbonate supplementation during 4 weeks of high-intensity interval training (8 sessions) did not positively impact outcomes such as rowing time, peak power, and mean power [29]. Another study explored the effect of sodium bicarbonate supplementation during 10 weeks of resistance training and did not find an ergogenic effect on 1RM strength [108]. However, in both studies that did not find an ergogenic effect, the sample sizes were very small (i.e., 4 to 6 participants per group), which resulted in a low statistical power [29, 108]. Overall, there is some evidence that the repeated use of sodium bicarbonate supplementation (0.2 to 0.4 g/kg ingested 90 to 60 min before every exercise session for 6 to 8 weeks) may impact long-term adaptations to exercise (i.e., delaying time to fatigue and increasing peak power), but much more work in the area is needed.

### Sex-specific effects of sodium bicarbonate on exercise performance

A recent systematic review [199] showed that only around 20% of studies examining the effects of sodium bicarbonate involved women as participants. Of these studies, several reported an ergogenic effect of sodium bicarbonate. For example, two studies by Bishop et al. [7, 8] reported that sodium bicarbonate improved repeated-sprint and intermittent sprint performance. While others reported similar findings [24, 99], some studies [62] did not find an ergogenic effect of sodium bicarbonate. According to the meta-analytical data of Saunders et al. [199], only 11 studies provided group analyses exclusively for women. When the results of these studies were pooled in a meta-analysis, an average increase in plasma bicarbonate following sodium bicarbonate ingestion was found to be around 7 mmol/L. The review also indicated that sodium bicarbonate is ergogenic in females with small-to-moderate exercise performance improvements (pooled effect size = 0.37). Nonetheless, the vast majority of studies that have explored (and established) the ergogenic effects of sodium bicarbonate on exercise performance included men only as participants. It currently seems that sodium bicarbonate is ergogenic for both sexes; however, the evidence base for a performance-enhancing effect in women is not as large. Although menstrual cycle phase has been shown to have very little influence on exercise outcomes [200], the size of some differences between phases represents >30% of the ergogenic effects shown in this meta-analysis (effect size 0.14 vs. 0.37), meaning it might be an important factor. Further work with female participants is required to determine the influence of the menstrual cycle phase on exercise outcomes following sodium bicarbonate supplementation.

### Training status and the effects of sodium bicarbonate on exercise performance

Studies that examined the ergogenic effects of sodium bicarbonate supplementation on exercise performance included different populations, ranging from elite athletes to untrained individuals (Table 1). Accordingly, training status could be a variable moderating the effects of sodium bicarbonate on exercise performance. Studies included well-trained basketball players [2], judo competitors [4], distance runners [6], team-sport athletes [7, 8], varsity track athletes [10], resistance-trained individuals [16], well-trained cyclists [27], hockey players [33], professional boxers [49], elite swimmers [67], taekwondo black belt athletes [68], and triathletes [86], and all reported an ergogenic effect of sodium bicarbonate on exercise outcomes. Studies have also included untrained participants and reported an ergogenic effect of this supplement [18, 19, 57, 73, 96, 97, 109]. Therefore, the absence of an ergogenic effect in some studies does not seem to be related to the training status of included participants. However, the studies analyzed herein also differed in a range of methodological characteristics that may have affected the effect sizes independent of the training status (e.g., exercise test, sodium bicarbonate dose, and timing of ingestion). Therefore, future studies should consider including participants with different training levels (e.g., untrained, trained, competitive, and elite athletes) to determine directly whether the effects of sodium bicarbonate vary according to training status.

### Optimal protocols of sodium bicarbonate supplementation

#### Powder and capsule form of sodium bicarbonate

In recent years, studies generally provided sodium bicarbonate supplementation to participants in capsule form. This form of supplementation is used to maintain a double-blind study design. Still, some of the early studies [6, 18, 19, 41, 42, 44, 77] mixed sodium bicarbonate with water (with or without additional substances such as orange juice or low-calorie sweetener), and this solution was ingested before exercise. There are several limitation with the latter approach. The taste of sodium bicarbonate is bitter and salty, which many participants may find unpleasant. In addition, due to its taste, it is difficult to disguise the sodium bicarbonate condition with this form of ingestion, which may compromise blinding. Still, a limitation with capsules is that a large number of them need to be ingested to get to ergogenic doses of sodium bicarbonate. Therefore, readers are also advised to consider the form of sodium bicarbonate supplementation used in a given study when interpreting its findings.

#### Sodium bicarbonate dose

Because the dose of sodium bicarbonate most commonly used in research trials is 0.3 g/kg, the evidence base for the ergogenic effects of sodium bicarbonate is most established for this dose. The origin for this protocol likely stems from the work of Jones et al. [58]. Additional support for the use of this dose can be found in one of the most cited dose-response studies on the topic, published by McNaughton in 1992 [77]. This study explored the effects of consuming sodium bicarbonate in incremental doses of 0.1 g/kg (from 0.1 to 0.5 g/kg) on peak power and total work produced during 60 s of high-intensity cycling. Increases in total work were observed with doses from 0.2 to 0.5 g/kg, and for peak power, doses from 0.3 to 0.5 g/kg were required to record an improvement. No significant differences in ergogenic effects were found between doses from 0.3 to 0.5 g/kg. However, side-effects were higher with 0.4 and 0.5 g/kg; thus, it was concluded that the dose of 0.3 g/kg provides an optimal cost/benefit balance [77].

Other studies have also investigated the effects of consuming different doses of sodium bicarbonate on exercise performance [38, 46, 47, 55, 75, 99, 121]. The smallest researched dose was 0.1 g/kg. A study by Ferreira et al. [38] compared the effects of consuming 0.1 g/kg vs. 0.3 g/kg of sodium bicarbonate on performance in a cycling test to exhaustion. Time to fatigue was improved only following the ingestion of 0.3 g/kg (76 ± 4 s cycling). The dose of 0.1 g/kg was not ergogenic, and the average performance value with this dose was very similar to the placebo condition (65 ± 8 s and 68 ± 5 s, respectively). Similar findings were reported in another study [99] that compared the effects of consuming 0.1 vs. 0.2 g/kg of sodium bicarbonate on repeated cycling in 12 moderately trained females. Time to exhaustion increased following the ingestion of 0.2 g/kg (162.4 ± 107.3 s), compared with 0.1 g/kg (133.9 ± 83.3 s) and a placebo (129.4 ± 104.0 s). There were no significant differences between the lower dose of sodium bicarbonate and placebo. Another study used doses of 0.1, 0.15, and 0.2 g/kg [55]. While the dose of 0.1 g/kg was not found to be ergogenic, there was also a general absence of improvements in performance (4 × 2-min cycling sprints) following sodium bicarbonate ingestion at any dose. Still, it is unclear whether the doses or some other element of the study protocol led to the absence of significant effects. It currently seems that a sodium bicarbonate dose of 0.1 g/kg is not ergogenic for exercise performance. The reason for this lack of an ergogenic effect might be that increases in plasma bicarbonate concentration following the ingestion of 0.1 g/kg of sodium bicarbonate are very small (1 to 2 mmol/L) [55, 201].

A dose of 0.15 g/kg has also been used, albeit only in two studies [55, 75]. One study [75] reported ergogenic effects with 0.15 g/kg and 0.3 g/kg of sodium bicarbonate on repeated-cycling performance (5 × 1-min cycling with the sixth sprint to exhaustion), but no significant difference between the doses. The other study [55] used 2-min cycling sprints and evaluated total work as the performance outcome following the ingestion of 0.1 g/kg, 0.15 g/kg, and 0.2 g/kg, although none of the doses were shown to be ergogenic. Thus, the efficacy of a 0.15 g/kg dose remains unclear.

Several studies have reported an ergogenic effect with a dose of 0.2 g/kg [19, 43, 57, 117]. Costill et al. [19] used this dose in their study that reported an improvement in time to exhaustion in a repeated-cycling test, coupled with substantial increases in plasma bicarbonate concentration of 5 mmol/L. In addition to McNaughton’s previously discussed work [77], several other studies compared the effects of 0.2 g/kg and 0.3 g/kg of sodium bicarbonate [46–48, 50, 51, 121]. Most studies reported an ergogenic effect for both doses, with no significant differences in the effects between the doses [46, 48, 50, 51, 121]. However, one study [47] reported that performance in a 4-km cycling time trial was improved only following the ingestion of 0.3 g/kg. Examination of individual responses in one study indicated that a dose of 0.2 g/kg was ergogenic in 8 out of 12 participants, while the dose of 0.3 g/kg was ergogenic for 11 out of 12 participants [51]. The difference between the percentages of participants whose performance improved following the ingestion of 0.2 g/kg and 0.3 g/kg of sodium bicarbonate was not statistically significant (*p* = 0.132). Future research is needed to clarify any potential differences between ergogenic effects of consuming 0.2 g/kg and 0.3 g/kg of sodium bicarbonate.

In summary, the following is currently known regarding the dose of sodium bicarbonate: (1) a dose of 0.1 g/kg does not appear to be ergogenic for exercise performance; (2) a dose of 0.15 g/kg may be the minimal ergogenic dose, albeit this was found only in one out of two studies; (3) in several studies, a dose of 0.2 g/kg was ergogenic, with some showing similar effects to those with higher doses; (4) a dose of 0.3 g/kg has been the most thoroughly studied dose of sodium bicarbonate, it has consistently produced ergogenic effects and is considered as the optimal dose; (5) doses of 0.4 g/kg and 0.5 g/kg are also ergogenic, but they do not seem to be a requirement to elicit improvements in exercise performance and are associated with a higher incidence and severity of side-effects.

Of note, this section reviewed the evidence and provided recommendations only for single-dose protocols of sodium bicarbonate ingestion. However, the optimal dose for multiple-day sodium bicarbonate supplementation protocols might be higher (see the section “Multiple-day protocols of sodium bicarbonate supplementation”).

#### Timing of sodium bicarbonate supplementation

In most studies, sodium bicarbonate was consumed at a standardized time point 60 to 180 min before exercise for all participants, making the evidence of its effectiveness around this timing of ingestion well established (Table 1). One of the determinants of the ergogenic effect of sodium bicarbonate may be the increase in plasma bicarbonate concentration [202]. It has been suggested that to increase the likelihood of an ergogenic effect, the start of the exercise session should coincide with the peak concentration of plasma bicarbonate concentration or with an increase in plasma bicarbonate concentration of 5 mmol/L [202]. However, there is a considerable variation between individuals in time from ingestion of sodium bicarbonate to the peak plasma bicarbonate concentration. For example, in one study [201], time to peak plasma bicarbonate concentration varied highly between individuals, as it occurred from 30 to 150 min, 40 to 165 min, and 75 to 180 min after the ingestion of sodium bicarbonate in the doses of 0.1, 0.2, and 0.3 g/kg, respectively.

The large individual variability in time from sodium bicarbonate ingestion to the peak plasma bicarbonate concentration may have presented a methodological issue in previous studies on ergogenic effects of this supplement using a standardized ingestion time. An alternative approach, incorporated into several recent studies involves sodium bicarbonate ingestion according to individualized considerations of time to peak plasma bicarbonate concentration [9, 22, 23, 46, 47, 50, 84]. In such studies, time to peak plasma bicarbonate concentration following sodium bicarbonate supplementation was first evaluated under resting conditions. Then, sodium bicarbonate intake is adjusted for the exercise trials according to individual timing at which peak plasma bicarbonate occurred. While the time to peak value is typically 60 to 70 min across individuals, this can vary from 10 min to 240 min after ingestion [22, 23, 46, 47, 50, 53, 84, 201, 203]. When using such an ingestion protocol, most studies showed ergogenic effects of sodium bicarbonate. For example, one study [84] used a repeated-sprint ability protocol (10 × 6-s sprints with 60-s rest) and reported that ingesting 0.3 g/kg according to individualized time to peak plasma bicarbonate concentration increased total work across all 10 sprints. The study by Boegman et al. [9] is the only one to date that compared the effects of individualized vs. non-individualized ingestion timing and reported that the former protocol produced greater ergogenic effects, although this study was limited by a lack of a placebo trial. It showed that individualized timing produces larger ergogenic effects than supplementation at a standardized pre-exercise time point (60 min), although this time point might be on the lower end of the optimal standardized timeframe [203]. Supplementation protocols that utilize individualized ingestion timing provide substantial practical difficulties because most athletes do not have access to a blood gas analyzer that would be required to determine peak plasma bicarbonate concentration and individualize supplementation timing.

Despite the generally favorable effects of individualizing ingestion timing, some have questioned the reliability of time to peak plasma bicarbonate concentration following sodium bicarbonate supplementation [203]. Oliveira and colleagues used three repeated administrations of sodium bicarbonate and reported that the time to peak plasma bicarbonate concentration was inconsistent and non-reproducible [203]. This study also suggested that ingesting a dose of 0.3 g/kg produces a long-lasting ergogenic potential window that lasts around 3 h, starting around 60 min after ingestion, when considering increases of plasma bicarbonate concentration >5 mmol/L [203]. Indeed, studies providing sodium bicarbonate supplementation at 60 min, 90 min, 120 min, 150 min, or 180 min before exercise have reported ergogenic effects [4, 7, 19, 25, 58, 102]. Based on these results, we conclude that sodium bicarbonate can be ergogenic when ingested from 60 to 180 min before exercise, but individual experimentation around timing is recommended, particularly concerning the incidence of side-effects.

When deciding about the timing of sodium bicarbonate ingestion, possible side-effects and their interference with the activity should also be considered (see section “Side-effects associated with sodium bicarbonate supplementation”). The decision to use or not to use individualized ingestion timing may also depend on the dose of sodium bicarbonate. Specifically, lower doses of sodium bicarbonate (e.g., 0.2 g/kg) result in shorter increases in plasma bicarbonate concentration [77]. However, when using higher doses of sodium bicarbonate, such as 0.3 g/kg, the increases in plasma bicarbonate concentration are longer-lasting [203]. Therefore, individualized ingestion timing may be more important when using lower doses of sodium bicarbonate. More dose-response studies are needed to confirm this hypothesis.

Finally, when deciding about the timing of sodium bicarbonate ingestion, the size of the capsules may also need to be considered. A recent study reported that peak plasma bicarbonate concentration following the ingestion of 0.3 g/kg of sodium bicarbonate occurred at 94 ± 24 min, 141 ± 27 min, and 121 ± 29 min when using small, medium and large size capsules, respectively [204]. For those aiming to increase extracellular buffering capacity more quickly, these recent results suggest that supplementation with sodium bicarbonate in smaller capsules should be considered [204].

#### Multiple-day protocols of sodium bicarbonate supplementation

Most studies have used protocols in which sodium bicarbonate is ingested acutely, from 60 to 180 min before exercise. However, several studies [15, 24, 26, 27, 31–34, 59, 66, 81–83, 88, 147] have provided supplementation for several consecutive days before testing exercise performance, ceasing on the evening before the trial. This approach is interesting because it could help avoid potential sodium bicarbonate-induced side-effects on the day of competition. Specifically, in these studies, sodium bicarbonate is provided daily for several days before the exercise test, with the last dose commonly ingested on the night before evaluating exercise performance. In most of these studies, there was no sodium bicarbonate ingestion on the day of the exercise test. For example, in one study [27], sodium bicarbonate was provided for three consecutive days before testing, with the last dose ingested at 19:00 h on the night before testing. The multiple-day protocol was initially used by McNaughton et al. [81], who provided an overall dose of 0.5 g/kg per day (split into four smaller doses to be ingested throughout the day) to their participants for 5 days before evaluating performance in a 60-s cycling test on the 6^th^ day (i.e., the day after the final ingested dose). This supplementation protocol was effective for improving total work (~14% increase) and peak power (~10% increase) during the exercise task.

Whilst there are no likely benefits of ingesting doses higher than 0.3 g/kg for single-dose supplementation protocols, multiple-day supplementation protocols may require a larger daily sodium bicarbonate dose to produce an ergogenic effect. One dose-response study [26] evaluated performance in the Wingate test following 5 days of supplementation with either 0.3 or 0.5 g/kg per day of sodium bicarbonate and found increases in mean power only with the higher dose. Other studies [59, 66] that used multiple-day supplementation (lasting from 3 to 7 days) also did not show significant ergogenic effects with a 0.3 g/kg per day dose. In contrast, most other studies [24, 26, 27, 81–83, 88, 147] that provided supplementation for 3 to 7 days with doses of 0.4 or 0.5 g/kg per day reported ergogenic effects of sodium bicarbonate. Therefore, for protocols that last between 3 and 7 days, it seems that a dose of 0.4 or 0.5 g/kg per day is needed to elicit an ergogenic effect. Still, it should be mentioned that a research group recently conducted a series of studies [31–34] in which much smaller doses of sodium bicarbonate were ingested over 10 days, with progressive increases in the doses (i.e., daily doses ranged from 0.025 g/kg per day to 0.100 g/kg per day). These studies reported ergogenic effects on mean power, muscular endurance, and the number of throws in a dummy throw test. This would suggest that a lower dose (i.e., up to 0.100 g/kg per day) of sodium bicarbonate ingested in a multiple-day protocol may also be ergogenic, at least when ingested for 10 days. However, future studies replicating this protocol of ingestion are needed to confirm the findings.

While it is clear that multiple-day sodium bicarbonate supplementation protocols can be effective, it is unclear how their effects compare to those observed following single-dose ingestion of sodium bicarbonate. A meta-analysis [205] included studies that provided sodium bicarbonate supplementation for 5 to 7 days before a Wingate test [26, 88, 147]. In this analysis, an ergogenic effect was found for mean power. This same meta-analysis, when focusing on studies that provided single-dose supplementation, did not find a significant ergogenic effect of sodium bicarbonate on performance in single and repeated Wingate tests [205]. Still, none of the studies included in this meta-analysis directly compared these two protocols of supplementation, so no inferences can be made on which (if any) of the protocols are more effective.

In two studies [15, 59] that compared the effects of single-dose vs. multiple-day protocols of sodium bicarbonate ingestion, no significant ergogenic effect was found for any of the two protocols. In another study, both protocols were comparably ergogenic [27]. One study [33] reported that only a multiple-day ingestion protocol was ergogenic for mean and peak power in the Wingate test. In the same study, both protocols elicited an ergogenic effect on performance in a hockey-specific field performance test [33]. Finally, McNaughton and Thompson [83] evaluated exercise performance during 90 s cycling after ingesting 0.5 g/kg 90 min before exercise and after ingesting 0.5 g/kg for 5 days before exercise. Exercise performance was evaluated on three consecutive days. On the second and third testing day, there was no sodium bicarbonate supplementation. Both ingestion protocols increased the total work in the cycling task on the first testing day. However, only the multiple-day protocol was ergogenic on the following two days. This suggests that the improvement in performance with this supplementation protocol may be longer-lasting. These results suggest that sodium bicarbonate loading may benefit athletes who have competition events scheduled on consecutive days. However, given that the mechanism underpinning this ergogenic effect is unclear, these findings should be taken with caution and they require further confirmation.

A final point to consider is that multiple-day sodium bicarbonate supplementation protocols require ingestion of a high number of capsules for several days, which may result in a continuous sensation of satiety [206]. This may, at least hypothetically, prevent athletes from meeting their daily energy demands and thus has the potential to negatively affect their performance. To circumvent this, athletes may consider using powder form of sodium bicarbonate mixed with liquid, even though there are limitations with this approach as well (see section “Powder and capsule form of sodium bicarbonate”).

#### Influence of warm-up on the ergogenic effects of sodium bicarbonate

The influence of different warm-up strategies on the ergogenic effects of sodium bicarbonate has been explored in two studies. Gurton et al. [207] explored whether an intermittent, sprint-based warm-up relative to lactate threshold (5 min at 50%; 2 min at 60%; 2 min at 80%; 1 min at 100%; 2 min at 50%; and 3 × 10-s maximal sprints with 90-s recovery) impacted the ergogenic effects of individualized sodium bicarbonate ingestion on 4-km cycling time-trial performance compared to a control warm-up (16.5 min cycling at 150 W). Their results showed that the high-intensity warm-up mitigated the ergogenic effects of sodium bicarbonate in club-level cyclists, likely due to the use of the increased buffering capacity during this intense warm-up. More recent data [208] reported that a high-intensity warm-up relative to maximal power output (5 min at 60% maximal power output, 5 min at 70% maximal power output, 5 min at 80% maximal power output, and 30 s at maximal power output) did not modulate the ergogenic effect of sodium bicarbonate on exercise capacity during a cycle to exhaustion tasks compared to a low-intensity warm-up (15-min cycling at 60% maximal power output). The difference between these studies may be related to the time between the warm-up and the exercise bout. Specifically, Gurton et al. [207] allowed 10 min between the end of the warm-up and the exercise bout, while Jones et al. [208] used 30 min; both studies cited personal experience in elite sport for these periods. The 30 min recovery period allowed for a substantial rebound effect in blood variables, leading to higher pre-exercise blood values with sodium bicarbonate regardless of exercise intensity, whereas the 10-min recovery period led to lower blood values following the high-intensity warm-up compared to the low-intensity warm-up. Thus, athletes may wish to leave sufficient recovery time between the end of the warm-up and the start of the main exercise event to ensure blood variables have returned to levels from which they will benefit. Further work should determine how much different recovery periods influence the ergogenic effects of sodium bicarbonate and whether these effects are seen across different modalities (e.g., running, swimming).

#### Sodium bicarbonate supplementation for multiple events

In many sporting events that would theoretically benefit from sodium bicarbonate ingestion, competition outcomes are decided through a series of heats and finals (e.g., 4000-m track cycling pursuit, rowing events) or via multiple events (e.g., events within the decathlon and modern pentathlon). Besides, in some sports, athletes may compete in more than one event in the same competition program (e.g., a swimmer may compete in several individual events and relays). In specific scenarios, including those identified above, the interval between bouts/events is often between 1 and 24 h, and the subsequent event may fall within the half-life of the changes in plasma bicarbonate concentrations achieved via sodium bicarbonate supplementation or before the body’s return to normal physiological status following the preceding event. One study [59] explored the effects of sodium bicarbonate on 200-m swimming time (measured on two consecutive days) in eight highly trained male swimmers. This study compared the effectiveness of single-dose and multiple-day supplementation protocols, neither of which was continued on the day of the second performance test. Such intervention design mimicked a real-life swimming competition timetable, with repeated races held 24 h apart. This study did not find a significant ergogenic effect, despite attempting to simulate an event known to cause acidosis [59]. This study did not provide a detailed time course of changes in plasma bicarbonate concentration over the period from the last dose to the second trial, which would be essential information for assessing their protocol and informing future similar studies. Therefore, future research is needed to explore the effects of sodium bicarbonate for multiple events.

### Side-effects associated with sodium bicarbonate supplementation

It is important to consider potential side-effects associated with sodium bicarbonate supplementation, including gastrointestinal discomfort. The buildup of CO_2_ in the gut, resulting from supplementation with sodium bicarbonate (Figure 1), may cause bloating, nausea, vomiting, and abdominal pain [209]. Furthermore, the incidence and severity of these side-effects increase linearly with the dose of sodium bicarbonate ingested and should be considered in terms of their overall effect on performance [77]. Additionally, it should be mentioned that long-term sodium bicarbonate supplementation at commonly employed doses (0.3 g/kg) will lead to an increased habitual sodium load for the body, which may contribute to exceeding the Tolerable Upper Intake Level for sodium specified in dietary guidelines. However, the effects of sodium bicarbonate supplementation on kidney function are not yet known and are deserving of investigation.

A 1995 study by Bird et al. [6] reported an ergogenic effect of sodium bicarbonate on 1500-m running. However, scrutiny of the individual responses revealed that two participants did not improve their running performance following sodium bicarbonate ingestion. Both of these participants experienced side-effects, such as stomachache and diarrhea, suggesting that these adverse reactions may have influenced their race times. Accordingly, the absence of an ergogenic effect in some studies might be due to the side-effects associated with sodium bicarbonate. Indeed, adverse gastrointestinal effects negated the performance benefits from sodium bicarbonate supplementation in the study by Saunders et al. [98]. They found a significant overall performance improvement only after excluding four participants who experienced gastrointestinal discomfort (from the total sample of 21 participants). The potential for side-effects to diminish the ergogenic effect of sodium bicarbonate was confirmed in a subsequent study from the same research group [40]. However, not all studies had shown a significant impact of side-effects on ergogenic effects of sodium bicarbonate, at least when the side-effects tended to be less severe (e.g., minor gastrointestinal distress) [95]. Unfortunately, many studies, especially the older ones, did not provide a detailed and comprehensive record of side-effects, making it difficult to determine how much this may have contributed to the lack of ergogenic effects of sodium bicarbonate in some studies. Therefore, future studies that examine the ergogenic effects of sodium bicarbonate should aim to also record and report side-effects associated with this supplement.

Reducing or eliminating the side-effects of sodium bicarbonate ingestion will likely increase its ergogenic effects. To accomplish this, several strategies can be considered. One is related to the timing of ingestion. Siegler et al. [103] reported lower gastrointestinal discomfort 180 min after supplementation, compared to 60 and 120 min after supplementation, even though the increase in plasma bicarbonate concentration was similar between the conditions. Carr et al. [210] reported that the greatest incidence of side-effects occurred 90 min after ingestion, suggesting that conducting performance testing at this time might not be optimal. Therefore, merely adjusting the timing of sodium bicarbonate ingestion relative to exercise may reduce the incidence and severity of side-effects that occur during the exercise.

Another option to minimize side-effects is to ingest sodium bicarbonate alongside a meal. Compared to protocols that involved isolated sodium bicarbonate ingestion, combining sodium bicarbonate (0.3 g/kg) with a high-carbohydrate meal (bread with fruit spread and cereal bars, including 1.5 g of carbohydrate per kilogram of body weight) resulted in alkalosis coupled with the lowest incidence of gastrointestinal symptoms [210]. This may be an optimal strategy for athletes who consume a high-carbohydrate meal within a few hours of training or competition. Additionally, these results highlight the potential importance of controlling pre-trial diet when exploring the effects of sodium bicarbonate supplementation. As mentioned previously, using lower doses of sodium bicarbonate (e.g., 0.2 g/kg) may be another strategy to reduce the incidence and severity of side-effects (see section “Sodium bicarbonate dose”).

More recent evidence suggests that the use of enteric-coated capsules may minimize side-effects by avoiding the interaction with stomach acid [53]. Such capsules can be used for acid-sensitive ingredients, such as sodium bicarbonate, to bypass the stomach [53]. As shown in a proof-of-principle case study [211] that included a post-bariatric surgery participant, bypassing the stomach led to some of the greatest increases of plasma bicarbonate concentration ever reported, of 12 to 20 mmol/L, while minimizing the associated side-effects. Hilton et al. [53] compared the effects of sodium bicarbonate ingestion in a dose of 0.3 g/kg in enteric-coated or gelatin capsules on performance in 4-km cycling time trials. The time required to complete 4 km of cycling was reduced following the ingestion of sodium bicarbonate in both enteric-coated and gelatin capsules (8.5 s and 9.6 s, respectively). Out of 11 participants, only three experienced gastrointestinal discomfort after ingesting enteric-coated capsules, compared to seven after ingesting gelatin capsules. Given the relatively small sample size in this study, more research is needed to draw solid conclusions about the possible reduction in the incidence and severity of side-effects following the ingestion of sodium bicarbonate in enteric-coated capsules. Finally, it should be considered that enteric-coated capsules can potentially prevent gastric symptoms, but they are unlikely to prevent intestinal side-effects such as osmotic diarrhea.

In summary, the most common side-effects associated with sodium bicarbonate ingestion include bloating, nausea, and abdominal pain, and their incidence and severity increase with increasing dose. Importantly, experiencing these side-effects may negatively affect exercise performance and diminish the ergogenic effect of sodium bicarbonate. Ingesting sodium bicarbonate (i) in smaller doses (e.g., 0.2 g/kg or 0.3 g/kg), (ii) around 180 min before exercise or adjusting the timing according to individual responses to side-effects, (iii) alongside a high-carbohydrate meal, and (iv) in enteric-coated capsules, are possible strategies to minimize the likelihood and severity of side-effects. Finally, a prudent recommendation for athletes is to experiment with different sodium bicarbonate supplementation protocols during training to find their own “optimal” protocol with minimal side-effects, which they can later also use in competitions.

### Placebo effects associated with sodium bicarbonate supplementation

It is well-established that a favorable outcome can arise purely from believing that one has received a beneficial treatment [212]. Indeed, there is considerable evidence to support placebo effects in sport and exercise nutrition, including sodium bicarbonate supplementation. In a recent meta-analysis, Marticorena et al. [213] examined the effects of placebo ingestion compared to a non-placebo control condition (no substance ingestion) on exercise performance in studies involving supplementation of caffeine and buffering agents (including sodium bicarbonate). In this meta-analysis, placebo ingestion was found to provide a small but ergogenic effect (Cohen’s *d* = 0.09). Additional analyses from this review showed that around 30% of the performance-enhancing benefits of buffering agents could be attributed to the placebo effect [213]. A double-blind crossover design, in which all participants receive both the active treatment and placebo prior to performing an exercise task, is the study design most used when investigating the effect of sodium bicarbonate supplementation on exercise outcomes. Thus, the studies that do not include a non-placebo control session, in which no inert supplement is provided, may underestimate the total effect of the intervention. This is important because in the practical context a given individual will either *ingest* or *not ingest* the supplement (i.e., the deliberate use of a placebo is unlikely) [214].

To minimize the possibility of a placebo effect, studies generally attempt participant blinding (i.e., keeping participants unaware of the assigned treatment). While this is a recommended methodological approach, it is important to consider that some participants may discern between the sodium bicarbonate and placebo trials. Specifically, sodium bicarbonate supplementation may cause side-effects such as stomach cramps and nausea, which may enable some, but usually not all, participants to correctly identify the substance ingested. This observation was placed in context by the study of Higgins and Shabir [215], in which participants ingested a standard placebo (sodium chloride) or a sham placebo that aimed to mimic gut fullness and abdominal discomfort associated with sodium bicarbonate ingestion. The authors found that cycling at 100% of peak power output to exhaustion was improved by 9.5% (Cohen’s *d* = 0.27) following sham placebo ingestion. While significant, this improvement was smaller than the one observed in a study using the same exercise test, where sodium bicarbonate was actually ingested (17.5%; Cohen’s *d* = 0.50), likely because placebo ingestion does not alter physiological buffering capacity [52].

McClung et al. [216] conducted a similar study on the placebo effect of sodium bicarbonate supplementation. When giving the placebo pill to the participants, the researchers told them that the pill contains an ergogenic substance—sodium bicarbonate. The ingestion of placebo (alongside the misleading instruction) enhanced performance in 1000-m running time trials similar to the actual effect of sodium bicarbonate (1.5% and 1.7%, respectively). Therefore, while sodium bicarbonate may enhance performance due to physiological effects, some portion of the ergogenic effect may be purely due to outcome expectancy. Another possibility is that the placebo effect is similar to the effect of sodium bicarbonate on some exercise performance outcomes and for some supplementation protocols. Due to the possibility of a placebo effect, in studies that attempt participant blinding, researchers should evaluate the effectiveness of blinding and consider it when interpreting their findings [217]. Most of the available studies did not evaluate the effectiveness of blinding, which is a limitation that needs to be addressed in future research.

### Interaction of sodium bicarbonate with other ergogenic aids

Many athletes compete in events in which the use of several supplements may be justified to maximize performance gains [218]. This has important implications for how research findings are interpreted because most studies have investigated the isolated effects of ergogenic supplements on performance. Nonetheless, evidence is growing regarding combinations of supplements and whether they provide synergistic ergogenic effects or counteract each other. Studies have explored supplement combinations of sodium bicarbonate with other ergogenic aids including, beta-alanine, caffeine, creatine, and nitrates [1].

#### Sodium bicarbonate and beta-alanine

Concurrent use of sodium bicarbonate and beta-alanine is one of the most well-researched supplement combinations. Beta-alanine is an effective ergogenic aid that must be ingested over a longer-term (e.g., over several weeks) to increase muscle carnosine content, thereby increasing the buffering capacity of the muscle [219–222]. Given that beta-alanine increases intracellular pH-buffering and sodium bicarbonate increases extracellular pH-buffering, it appears reasonable to consider that combining the two would produce an additive ergogenic effect. The first study to investigate this showed a 12% improvement in high-intensity cycling capacity with beta-alanine, a 7% improvement with sodium bicarbonate, and a 16% improvement when concurrently using sodium bicarbonate and beta-alanine [144]. Other studies have also suggested potentially greater improvements when combining sodium bicarbonate and beta-alanine compared to sodium bicarbonate alone during a repeated 30-s arm cranking test [147], 2000-m rowing [138], and 100-m and 200-m swimming [133]. However, several studies have not shown significant additive benefits of co-supplementing these ergogenic aids [127, 132, 134, 146]. A meta-analysis showed that the addition of sodium bicarbonate to beta-alanine supplementation produced greater ergogenic effects than beta-alanine alone (pooled Cohen’s *d*: 0.43 vs. 0.18) [221]. Overall, the findings suggest that beta-alanine and sodium bicarbonate co-supplementation may generate potentially meaningful increases in exercise performance over supplementation with either alone.

#### Sodium bicarbonate and caffeine

Caffeine is a well-established ergogenic aid, with performance benefits reported for aerobic and muscular endurance, power, jump height, and muscular strength [223–229]. Caffeine’s ergogenic effects are generally explained by its ability to act as an adenosine receptor antagonist, which acts to reduce fatigue, pain, or perception of effort [228]. Given that sodium bicarbonate and caffeine could improve performance through different mechanisms, their concurrent use could produce additive effects. A recent review [230] reported that only one [135] out of eight studies on this topic showed additive effects of combining sodium bicarbonate and caffeine. Despite this, it should be considered that sodium bicarbonate supplementation protocols in some of the studies [137, 142] resulted in high incidence and severity of side-effects and were also not found to be effective. Therefore, it was concluded that further work is required to truly determine whether co-supplementation of caffeine and sodium bicarbonate produces larger ergogenic effects compared with supplementation with either of the supplements alone.

#### Sodium bicarbonate and creatine

Creatine is another well-researched ergogenic supplement [231]. Several studies explored the effects of using sodium bicarbonate and creatine concurrently and reported an additive effect. Co-supplementation with sodium bicarbonate (0.5 g/kg per day for 5 days) and creatine (20 g for 5 days) enhanced exercise performance (“Taekwondo Anaerobic Intermittent Kick Test”) in taekwondo athletes [143]. Another study involving competitive female and male swimmers provided 20 g of creatine for 6 days, followed by an acute dose of sodium bicarbonate in the dose of 0.3 g/kg on the morning of the seventh day. On the same day, repeated 100-m swim performance was evaluated [140]. Concurrent use of these supplements reduced the time needed to complete the second 100-m swim by 1 s (1.5%). Similarly, the combination of creatine and sodium bicarbonate increased peak and mean power and attenuated the decline in peak power to the greatest extent over repeated-sprints [126]. While only a handful of studies have been published on this topic thus far, the combination of sodium bicarbonate and creatine may provide superior benefits compared to ingesting sodium bicarbonate or creatine alone.

#### Sodium bicarbonate and nitrates

Due to their mechanisms of action, co-supplementation of sodium bicarbonate and nitrates may be counterproductive. Specifically, the alkalosis achieved by bicarbonate supplementation may decrease the effect of nitrate supplementation because the conversion of nitrate to nitric oxide is facilitated by an acidic environment [128]. Only one study to date has investigated the combined effect of these two supplements, with no significant effects on 4-km cycling time-trial performance found for nitrates (consumed through beetroot crystals) or sodium bicarbonate either in isolation or combined [128]. Given the paucity of studies investigating the effects of combining these supplements on exercise performance, no conclusions can be drawn at present. More research is needed on this topic.

### Position of the International Society of Sports Nutrition (ISSN)

Based on a comprehensive review and critical analysis of the literature regarding the effects of sodium bicarbonate supplementation on exercise performance, conducted by experts in the field and selected members of the International Society of Sports Nutrition (ISSN), the following conclusions represent the official Position of the Society:

1. Supplementation with sodium bicarbonate (doses from 0.2 to 0.5 g/kg) improves performance in muscular endurance activities, various combat sports, including boxing, judo, karate, taekwondo, and wrestling, and in high-intensity cycling, running, swimming, and rowing. The ergogenic effects of sodium bicarbonate are mostly established for exercise tasks of high-intensity that last between 30 s and 12 min.
2. Sodium bicarbonate improves performance in single- and multiple-bout exercise.
3. Sodium bicarbonate improves exercise performance in both men and women.
4. For single-dose supplementation protocols, 0.2 g/kg of sodium bicarbonate seems to be the minimum dose required to experience improvements in exercise performance. The optimal dose of sodium bicarbonate dose for ergogenic effects seems to be 0.3 g/kg. Higher doses (e.g., 0.4 or 0.5 g/kg) may not be required in single-dose supplementation protocols, because they do not provide additional benefits (compared with 0.3 g/kg) and are associated with a higher incidence and severity of adverse side-effects.
5. For single-dose supplementation protocols, the recommended timing of sodium bicarbonate ingestion is between 60 and 180 min before exercise or competition.
6. Multiple-day protocols of sodium bicarbonate supplementation can be effective in improving exercise performance. The duration of these protocols is generally between 3 and 7 days before the exercise test, and a total sodium bicarbonate dose of 0.4 or 0.5 g/kg per day produces ergogenic effects. The total daily dose is commonly divided into smaller doses, ingested at multiple points throughout the day (e.g., 0.1 to 0.2 g/kg of sodium bicarbonate consumed at breakfast, lunch, and dinner). The benefit of multiple-day protocols is that they could help reduce the risk of sodium bicarbonate-induced side-effects on the day of competition.
7. Long-term use of sodium bicarbonate (e.g., before every exercise training session) may enhance training adaptations, such as increased time to fatigue and power output.
8. The most common side-effects of sodium bicarbonate supplementation are bloating, nausea, vomiting, and abdominal pain. The incidence and severity of side-effects vary between and within individuals, but it is generally low. Nonetheless, these side-effects following sodium bicarbonate supplementation may negatively impact exercise performance. Ingesting sodium bicarbonate (i) in smaller doses (e.g., 0.2 g/kg or 0.3 g/kg), (ii) around 180 min before exercise or adjusting the timing according to individual responses to side-effects, (iii) alongside a high-carbohydrate meal, and (iv) in enteric-coated capsules are possible strategies to minimize the likelihood and severity of these side-effects.
9. Combining sodium bicarbonate with creatine or beta-alanine may produce additive effects on exercise performance. It is unclear whether combining sodium bicarbonate with caffeine or nitrates produces additive benefits.
10. Sodium bicarbonate improves exercise performance primarily due to a range of its physiological effects. Still, a portion of the ergogenic effect of sodium bicarbonate seems to be placebo-driven.

## Data Availability

Not applicable.

## Footnotes

1 Cohen's *d* effect sizes are commonly interpreted as: “trivial/very small” (<0.20), “small” (0.20–0.49), “medium” (0.50–0.79), and “large” (≥0.80)
